# Epidemiology and treatment of patients with iron deficiency and iron deficiency anemia: a study on longitudinal German health claims data

**DOI:** 10.1186/s12889-025-24730-9

**Published:** 2025-09-24

**Authors:** Karina C. Manz, Daniel Murphy, Flora Stavenow, Luis Guillermo Correa Astorquiza, Anja Cengia, Vukašin Višković, Ariane Höer, Anja Mocek

**Affiliations:** 1 IGES Institut GmbH, Friedrichstr. 180, Berlin, 10117 Germany; 2 Norgine Ltd, Uxbridge, UK; 3 Norgine GmbH, Wettenberg, Germany; 4 Norgine de España S.L.U., Madrid, Spain; 5 InGef – Institute for Applied Health Research Berlin GmbH, Berlin, Germany

**Keywords:** Iron deficiency, Anemia, Prevalence, Comorbidity, Therapeutics, Ferric maltol, Claims analysis

## Abstract

**Background:**

Iron deficiency (ID) and ID anemia (IDA), the most common deficiency diseases, may occur as isolated diseases or secondary to a causative disorder such as menorrhagia, inflammatory bowel disease (IBD), and heart disease (HD). As real-world data are limited, our objective was to assess the prevalence of ID/IDA, associated comorbidity, drug treatments focusing on trivalent ferric maltol, and healthcare resource utilization (HRU) with associated costs in Germany.

**Methods:**

This retrospective, non-interventional cohort study analyzed anonymized German health claims data covering 2016–2021. Data on patients with ID/IDA and matched control individuals, subgroups of patients with ID/IDA and concurrent disease (menorrhagia, IBD, HD), as well as patients with ID/IDA treated with ferric maltol were descriptively analyzed to evaluate prevalences, comorbidities (Elixhauser Comorbidity Index [ECI]), drug prescriptions, HRU, and HRU-associated costs.

**Results:**

In 2021, 129,462 individuals were diagnosed with ID/IDA. Concurrent menorrhagia, IBD, and HD were observed in 34,421, 8,422, and 44,699 individuals, respectively. ID/IDA prevalences were stable throughout the study at approximately 3.3% but were higher in older individuals (up to 10.1%) and women of reproductive age (5.6–7.35%). Comorbidity burden (ECI) was two-fold higher in patients with ID/IDA than in control individuals (4.5 ± 3.6 vs. 2.3 ± 2.5, 2021 [mean ± SD]) and increased with concurrent diseases (menorrhagia [3.4 ± 2.8], IBD [5.0 ± 3.9], HD [7.9 ± 3.5]). The most frequently prescribed medication was oral bivalent iron preparation (25.0–27.5%; parenteral iron: 5.8–6.5%; oral trivalent iron: 0.2–0.7%). Ferric maltol dispensations increased slightly from 2018 to 2021 compared to other trivalent iron preparations. In patients with ID/IDA, the percentages of hospitalizations and sick leaves were higher than in control individuals (41.2% vs. 22.0%; 21.4% vs. 3.1%), corresponding to higher total costs that further increased with concurrent disease. In the subgroup of patients treated with ferric maltol (*n* = 686), HRU was lower in some parameters, with slightly higher ID/IDA-related total costs. Within 180 days after treatment initiation, most patients continued ferric maltol and did not switch to another iron preparation.

**Conclusions:**

Despite the heterogeneity of the disease, monitoring prevalence of ID/IDA, comorbidities, drug treatments, and HRU may help identify populations at increased health risk to optimize treatment strategies.

**Supplementary Information:**

The online version contains supplementary material available at 10.1186/s12889-025-24730-9.

## Background

Iron deficiency (ID) is the depletion of total body iron, profoundly affecting central cellular functions such as DNA synthesis, oxygen transport, and red cell production. As iron is needed primarily for hemoglobin synthesis, anemia is an evident sign of iron deficiency, and iron deficiency anemia (IDA) is often considered synonymous with ID. At least 50% of anemia worldwide is thought to be due to ID [1]. ID/IDA is the most common deficiency disease in humans and one of the major contributors of global disease burden [2]. It is estimated that more than 1.2 billion individuals are affected by IDA, with wide variations from low-income to high-income regions [3]. No current precise data are available for Europe, but prevalence of ID/IDA in Europe is thought to be between 5% and 10% [4].

The main causes of ID/IDA include increased iron requirements of menstruating or pregnant women as well as children and adolescents due to rapid growth, low iron intake due to malnutrition, and decreased intestinal iron absorption due to gastrointestinal diseases and drug treatment. Other causes of ID/IDA are any chronic blood loss, which may result from, for example, menorrhagia, gastrointestinal lesions, and anticoagulant treatment, as well as the combination of chronic blood loss with decreased iron absorption in, for example, inflammatory bowel disease, and chronic heart failure [3]. Several of these conditions cannot only cause ID/IDA, but, conversely, are also aggravated by it. In heart failure, ID/IDA increases the risk of hospitalization and affects quality of life and survival [5].

Therapeutic iron supplementation is indicated for ID with manifested iron-deficient erythropoiesis and IDA. The route of administration and the choice of compound largely depend on clinical parameters, the underlying cause and its reversibility, as well as intolerances of patients. Oral bivalent iron (Fe^2+^) preparations such as ferrous sulphate, ferrous gluconate, and ferrous fumarate have traditionally been used to treat ID/IDA in a wide range of conditions [6]. However, a major difficulty with bivalent iron preparations is the poor intestinal absorption leading to adverse gastrointestinal effects and reduced patient compliance [7]. In Europe, parenteral iron substitution is indicated in patients who do not respond or are intolerant to oral iron as well as in patients requiring a rapid ID/IDA correction [8–10]. However, parenteral iron substitution is associated with higher health care costs, the inconvenience of intravenous infusion [11], and a risk of anaphylactic reactions [12].

In case of intolerance to bivalent oral iron preparations, trivalent (Fe^3+^) oral iron preparations such as ferric oxide polymaltose complexes and ferric maltol are increasingly considered as an alternative and are available for prescription in Germany among other countries [10, 13, 14]. After oral intake of ferric maltol, ferric iron reaches the intestinal mucosa in a complex form, which is likely to result in a more efficient uptake with improved tolerance [15]. Due to its beneficial effects on hemoglobin levels in patients and its favorable safety profile, ferric maltol has been approved for ID treatment in Europe [16] and has also been suggested an alternative to parenteral iron substitution in patients with inflammatory bowel disease [14].

Due to the different causes and consequences of ID/IDA, associated comorbidities, and treatment options, a better knowledge of the patients’ characteristics is required to develop and optimize treatment recommendations. However, real-world data on regional ID/IDA prevalences, the associated comorbidity burden, as well as the patterns of drug treatment and healthcare resource utilization (HRU) are limited.

We therefore assessed the prevalence of ID/IDA in Germany in a retrospective, non-interventional, matched cohort study, and characterized the population of patients with ID/IDA in terms of epidemiological and clinical factors, treatment patterns focusing on ferric maltol, as well as HRU and associated expenditures. Our study was based on claims data of patients with ID/IDA and a matched control population, covering an observational period from 2016 to 2021.

## Methods

### Study design

This study was a retrospective, non-interventional, matched cohort study using German claims data covering the period between January 1 st, 2016, and December 31 st, 2021. Anonymized claims data were provided by the Institute for Applied Health Research Berlin (InGef) sample database [17], an anonymized healthcare database containing claims data from a sample of four million individuals insured by the German statutory health insurance (SHI). Representativeness of the database for the German population has been demonstrated elsewhere [17]. The database provides demographic data, inpatient data (hospitalization with primary and secondary diagnoses, diagnostic and therapeutic procedures coded by the International Classification of Procedures in Medicine [German modification, ‘Operationen- und Prozedurenschlüssel’, OPS [18]), and outpatient data (diagnoses, outpatient services coded by the uniform value assessment of fee regulations, ‘Einheitlicher Bewertungsmaßstab’, EBM [19], drug prescriptions coded by the Anatomical Therapeutic Chemical [ATC] codes [20]). Diagnoses were coded using the German modification of the 10th version of the International Classification of Diseases (ICD-10-GM [21]). All data in the InGef database are anonymized and are no longer considered social data under German law (§ 67 para. 2 SGB X [Social Security Code X] and Art. 4 Nr. 1 GDPR [General Data Protection Regulation]). Consequently, the use of the database for health services research was fully compliant with German federal law and did not necessitate ethical review. Informed consent was not required as the study utilized anonymized administrative claims data that did not permit direct patient identification, and no contact was made with patients. This study was conducted in accordance with research practices described in the Good Epidemiological Practice (GEP) guidelines [22] and the Good Practice of Secondary Data Analysis (GPS) guidelines [23].

### Study populations

Individuals with continuous insurance coverage during at least one calendar year between 2016 and 2021 were eligible. Patients with ID/IDA were identified by an inpatient primary or secondary diagnosis or a confirmed outpatient diagnosis according to ICD-10-GM classification during each year of the observational period (codes: E61.1, D50.0, D50.8, D50.9). For each population of patients with ID/IDA, a matched control group without ID/IDA diagnosis (same age [± 4 years] and sex) was randomly selected from the study population at a ratio of three controls per case.

In addition, a subgroup of patients treated with ferric maltol (hereafter referred to as treatment cohort) was identified among patients with ID/IDA. Eligibility criteria were at least one dispensation of ferric maltol between 2017 and 2020 (index period), with an ID/IDA diagnosis within the same quarter and no dispensation with ferric maltol within one year before treatment initiation. The index date was the date of the first ferric maltol dispensation. Patients in the treatment cohort were observed for one year before and one year after the index date, thus the observation period for these patients ranged from 2016 to 2021.

Further subgroups were identified according to age, additional diagnoses or prescriptions for iron preparations documented within the same year of ID/IDA diagnosis (all patients) or within one year before and after the index date (treatment cohort). The following subgroups were considered due to their established associations with ID/IDA [3]:

(i) heart disease (ICD-10-GM: I05-I09, I20-I25, I27, I30-I52),
(ii) geriatric (age ≥ 70 years in the respective reference year or in the year of the index date),
(iii) menorrhagia or other gynecological diseases (ICD-10-GM: N80, N84-N85, N91-N93, N95; or prescription of iron supplementation claim by a gynecologist),
(iv) gastrointestinal bleeding (ICD-10-GM: C18-C20, D12, K25-K28, K31.82, K50-K52, K55.22, K62.1, K63.5), and.
(v) inflammatory bowel disease (ICD-10-GM: K50-K52).

### Procedures

The prevalence of ID/IDA was estimated and extrapolated to the total German population by age- and sex-standardized population estimates provided by the German Federal Statistical Office [24]. The comorbidity burden was categorized by the Elixhauser Comorbidity Index (ECI [25]) according to ICD-10-GM classification [26, 27]. In addition, the prevalence for specific diagnoses were assessed as defined in Additional file 1, Supplementary Table 1.

Drug prescriptions, HRU and associated costs, number and length of hospitalizations, and number and length of sick leaves were assessed for each population analyzed. Drug prescriptions were documented according to ATC-classification (provided in Table 3 of the results section), and hospitalizations and sick leaves were attributed to ID/IDA by the inpatient primary discharge diagnosis and sick note diagnosis, respectively. HRU associated costs were determined on the basis of SHI expenses. Inflation was taken into account by standardizing costs to 2021 on the basis of consumer price index data [28].

For the treatment cohort and subgroups, the mode of iron supplementation was documented in the year before and after the index period and categorized into five groups, namely no relevant treatment, bivalent oral iron, trivalent oral iron, parenteral iron, and iron supplementation with any of the aforementioned treatment options. Furthermore, any switch among treatments was documented. According to German guidelines, treatment with oral iron preparations should be continued for 4 to 6 months [4]. Therefore, prescription of another treatment option was considered as being a treatment switch if it occurred within 180 days after treatment initiation.

### Statistics

All analyses are descriptive and exploratory. Categorical variables were summarized by frequency and percentage, and continuous variables were presented as mean ± standard deviation (SD). All analyses were performed using the R Statistical Software [29].

## Results

### Data selection and patient flow

**Fig. 1:**
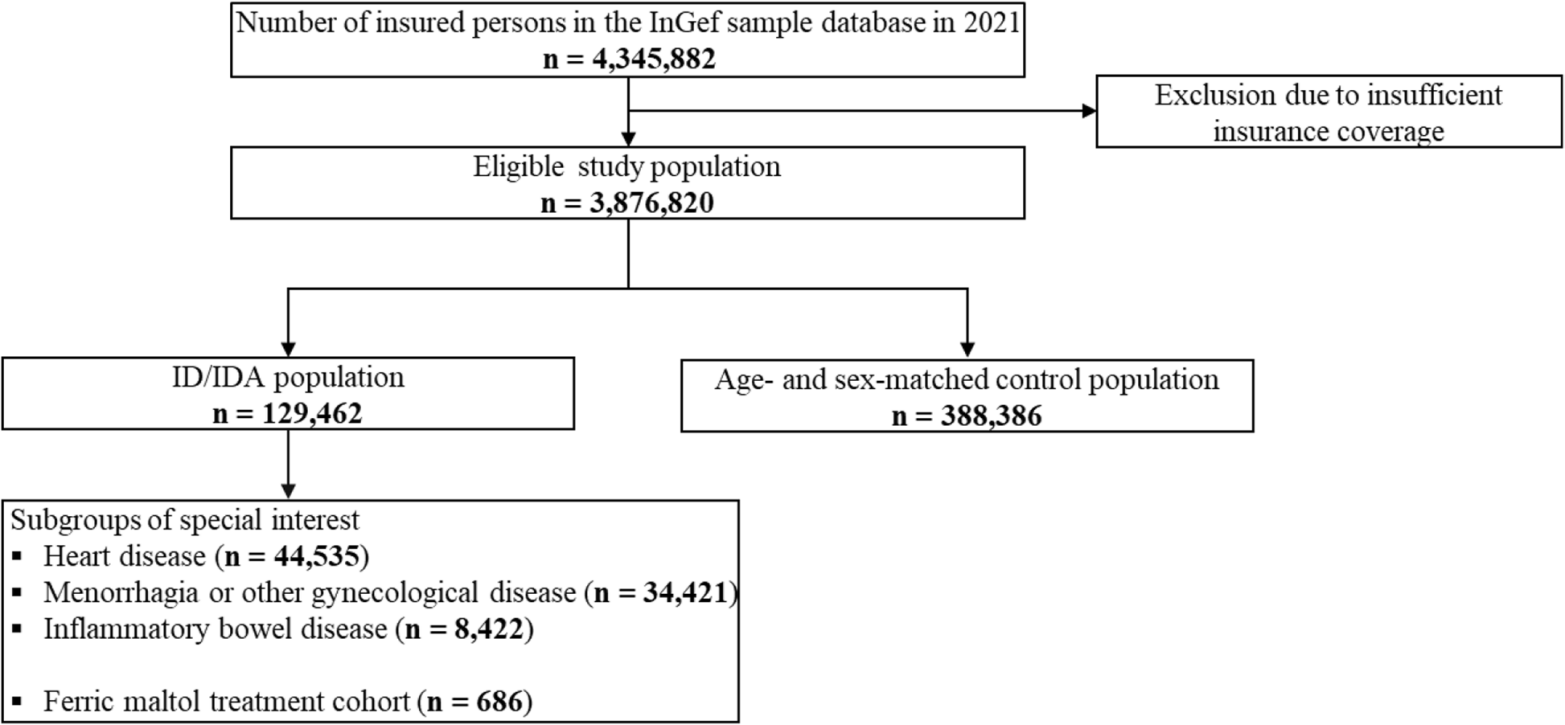
Flow of individuals through the study, exemplarily shown for 2021. Individuals were identified for each year of the study period (2016, 2017, 2018, 2019, 2020, 2021) to define study populations, except ferric maltol treated patients (treatment cohort; index period 2017–2020; ID/IDA: iron deficiency/iron deficiency anemia)

For each year of the observational period (2016–2021), study populations were identified, as shown for 2021 (Fig. 1).

In 2021, 129,462 individuals were diagnosed with ID and/or IDA, and subgroups of special interest were formed on the basis of additional criteria (heart disease, geriatric, menorrhagia or other gynecological diseases, gastrointestinal bleeding, inflammatory bowel disease) or ferric maltol treatment (number of individuals during the study period: see Additional file 1, Supplementary Tables 2–3).

### Prevalence

The resulting one-year prevalences of ID/IDA obtained from the database and extrapolated to the German population were stable over the study period at approximately 3.3% and 3.2%, respectively, corresponding to approximately 2.7 million individuals affected in Germany each year (Table 1).

**Table 1:** Estimated ID/IDA prevalence in the German population, 2016–2021

	InGef sample database Prevalence, % (*n*)	Extrapolation to the German population Prevalence, % (*n*)
2016	3.2% (128,746)	3.1% (2,593,718)
2017	3.3% (132,420)	3.2% (2,663,902)
2018	3.3% (132,179)	3.2% (2,683,168)
2019	3.4% (135,444)	3.3% (2,740,650)
2020	3.2% (129,716)	3.2% (2,636,295)
2021	3.3% (129,462)	3.3% (2,732,583)

*ID/IDA* iron deficiency/iron deficiency anemia

**Fig. 2:**
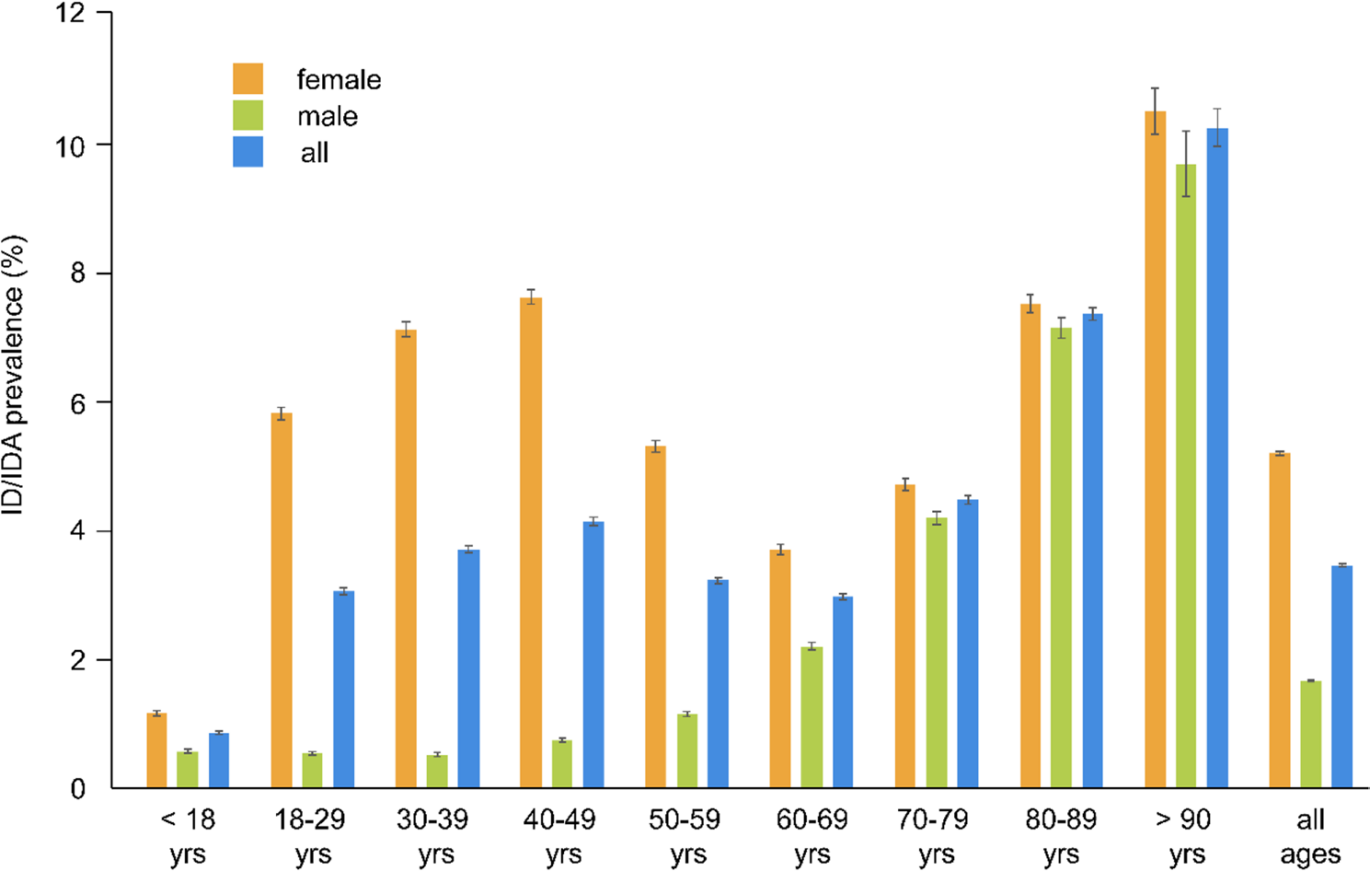
Prevalence of ID/IDA with 95% confidence interval in Germany, 2021 (ID/IDA: iron deficiency/iron deficiency anemia; yrs: years)

As shown for 2021, overall prevalence was higher in individuals with advanced age (< 18 years: 0.84% [95% CI, 0.82–0.86], >90 years: 9.87% [95% CI, 9.6–10.16]). Women were more affected than men, in particular in their reproductive age, with prevalences of 5.60%, 6.86%, and 7.35% in the age cohort of 18–29 years, 30–39 years, and 40–49 years, respectively (Fig. 2).

### Comorbidity burden

**Fig. 3:**
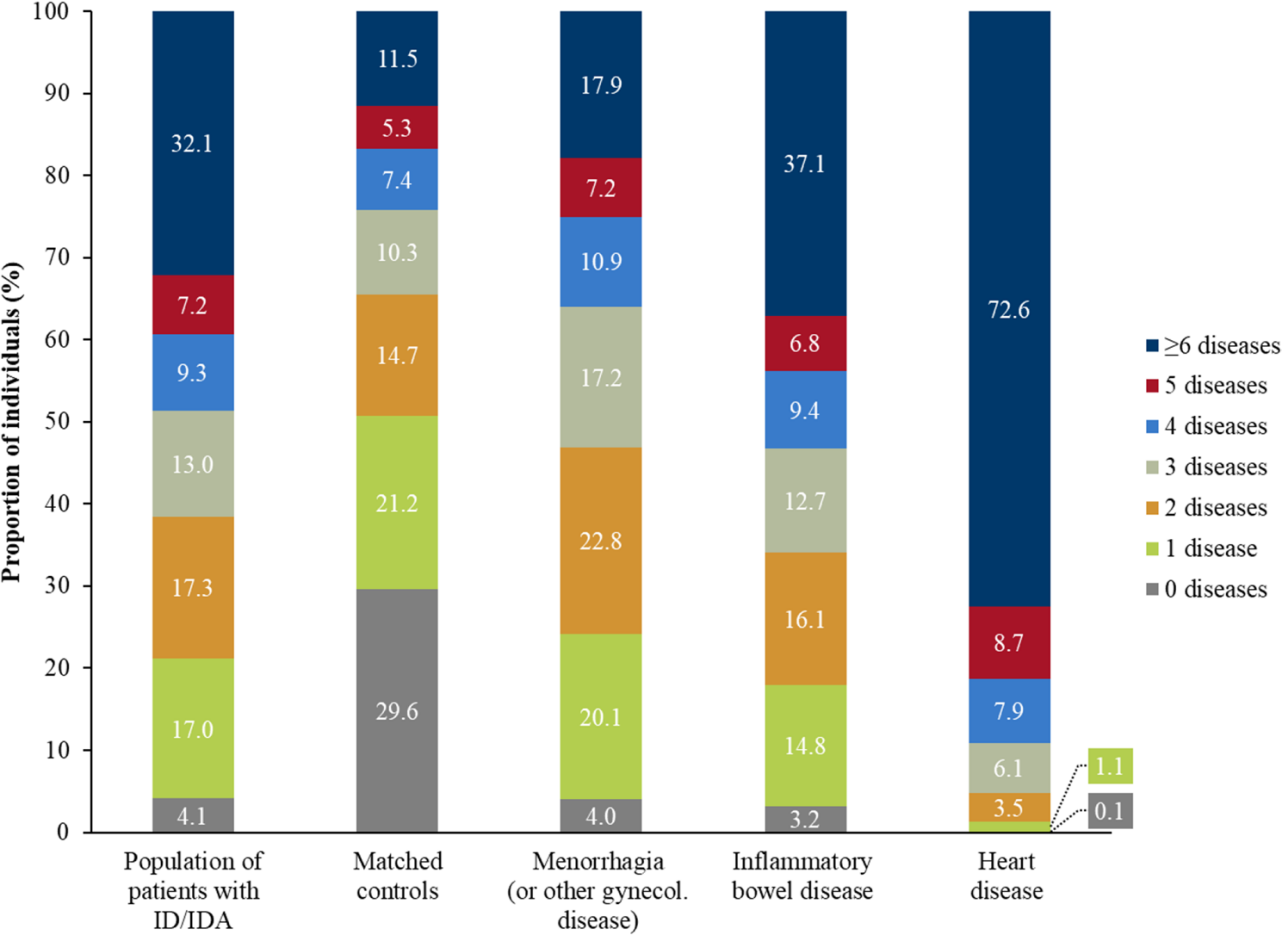
Comorbidity burden of patients with ID/IDA, matched control individuals, and patients with ID/IDA with concurrent diseases in Germany, 2021 (ID/IDA: iron deficiency/iron deficiency anemia)

In 2021, 32.1% of patients with ID/IDA and 11.5% of matched control individuals had six or more diseases of the ECI. When compared to control individuals, this comorbidity burden was further increased in patients with ID/IDA with concurrent menorrhagia or other gynecological diseases (17.9%), inflammatory bowel disease (37.1%), and heart disease (72.6%; Fig. 3).

During each year of the study period, the mean number of diseases of the ECI of the overall population of patients with ID/IDA was two-fold higher than that of matched control individuals (mean ± SD: 4.5 ± 3.6 vs. 2.3 ± 2.5 in 2021) and was higher among subgroups (mean ± SD: 3.4 ± 2.8 [menorrhagia or other gynecological diseases], 5.0 ± 3.9 [inflammatory bowel disease], and 7.9 ± 3.5 [heart disease]).

The most commonly observed pre-defined diagnoses in patients with ID/IDA with concurrent menorrhagia or other gynecological diseases were menopausal and other perimenopausal disorders (42.0%) and excessive, frequent and irregular menstruation (41.6%), while the most frequently observed pre-defined diagnoses in patients with ID/IDA with concurrent inflammatory bowel disease were noninfective gastroenteritis and colitis (61.1%), colitis ulcerosa (27.0%), and Crohn’s disease (25.7%). In patients with ID/IDA with concurrent heart disease, the most commonly observed pre-defined diagnoses were chronic ischemic heart disease (47.1%), heart failure (45.8%), and type 2 diabetes mellitus (40.6%) (Table 2).

**Table 2:** Prevalence of pre-defined diagnoses in patients with ID/IDA with concurrent menorrhagia, inflammatory bowel disease, and heart disease in Germany, 2021

Diagnosis	Prevalence (*n*)
Subpopulation: Heart disease	
Chronic ischemic heart disease	47.1% (20,968)
Heart failure	45.8% (20,397)
Type 2 diabetes mellitus	40.6% (18,070)
Atrial fibrillation and flutter	37.7% (16,772)
Gonarthrosis (arthrosis of knee)	25.2% (11,216)
Other cardiac arrhythmias	24.9% (11,095)
Nonrheumatic mitral valve disorders	24.7% (11,004)
Unspecified diabetes mellitus	21.5% (9,588)
Nonrheumatic aortic valve disorders	19.9% (8,839)
Other arthrosis	16.5% (7,355)
Subpopulation: menorrhagia or other gynecological diseases	
Menopausal and other perimenopausal disorders	42.0% (14,462)
Excessive, frequent and irregular menstruation	41.6% (14,317)
Pregnancy	14.9% (5,128)
Absent, scanty and rare menstruation	12.4% (4,270)
Gonarthrosis (arthrosis of knee)	11.3% (3,895)
Type 2 diabetes mellitus	10.7% (3,676)
Other arthrosis	8.7% (3,010)
Anemia complicating pregnancy, childbirth and the puerperium	7.9% (2,710)
Other abnormal uterine and vaginal bleeding	7.9% (2,709)
Other noninflammatory disorders of uterus, except cervix	7.4% (2,530)
Subpopulation: inflammatory bowel disease	
Other noninfective gastroenteritis and colitis	61.1% (5,147)
Colitis ulcerosa	27.0% (2,270)
Crohn’s disease	25.7% (2,164)
Type 2 diabetes mellitus	20.2% (1,699)
Heart failure	16.7% (1,410)
Chronic ischemic heart disease	16.0% (1,351)
Gonarthrosis (arthrosis of knee)	13.5% (1,135)
Atrial fibrillation and flutter	13.2% (1,114)
Unspecified diabetes mellitus	10.7% (904)
Menopausal and other perimenopausal disorders	10.5% (882)

*ID/IDA* iron deficiency/iron deficiency anemia

Mean number of diseases of the ECI ± SD throughout the study period as well as additional data on further subgroups are provided in Additional file 1, Supplementary Tables 4–5).

### Treatment of iron deficiency and iron deficiency anemia

**Fig. 4:**
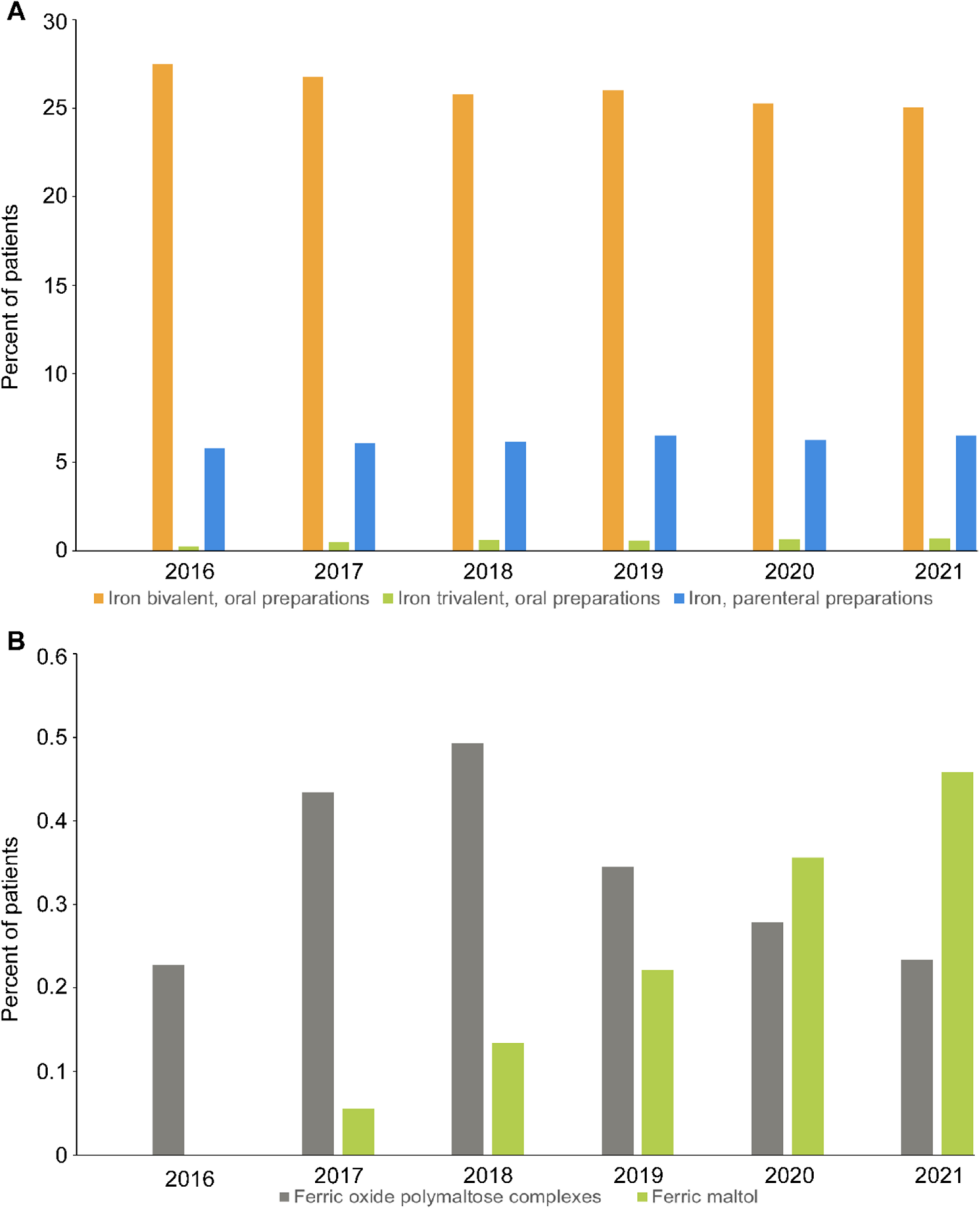
Usage of supplemental iron formulations in Germany, 2016–2021. **A** Percentage of patients with ID/IDA receiving oral bivalent, oral trivalent, and parenteral iron preparations during the study period. **B** Percentage of patients with ID/IDA receiving the oral trivalent the preparations ferric oxide polymaltose complex and ferric maltol (ID/IDA: iron deficiency/iron deficiency anemia)

Between 2016 and 2021, most of the patients with ID/IDA who were prescribed iron preparations received at least one dispensation for an oral bivalent iron preparation (25.0% – 27.5%), followed by parenteral (5.8% – 6.5%) and oral trivalent iron preparations (0.2% – 0.7%; (Fig. 4A).

Iron (Fe^2+^)-glycine sulfate and iron (Fe^2+^)-sulfate were the most widely used medications within the class of oral bivalent iron formulations, and ferric oxide polymaltose and ferric sodium gluconate complexes dominated the class of parenteral formulations. The ratio of medications within each class of formulations was almost unchanged over the study period (Table 3).

**Table 3:** Dispensations of supplemental iron preparations in Germany, 2016–2021 (patients with ID/IDA)

	Percentage of patients with ID/IDA (*n*)					
Class/agent (ATC-Code)	2016	2017	2018	2019	2020	2021
Iron bivalent, oral preparations (B03AA)	27.5% (35,383)	26.8% (35,470)	25.8% (34,108)	26.0% (35,199)	25.3% (32,773)	25.0% (32,420)
Iron (Fe^2+^) glycine sulfate (B03AA01)	20.8% (26,733)	20.3% (26,934)	19.8% (26,118)	19.8% (26,831)	19.5% (25,243)	19.4% (25,136)
Ferrous fumarate (B03AA02)	0.1% (165)	0.1% (172)	0.1% (152)	0.1% (156)	0.1% (136)	0.1% (140)
Iron (Fe^2+^) gluconate (B03AA03)	0.6% (757)	0.7% (899)	0.5% (643)	0.3% (474)	0.3% (392)	0.3% (367)
Iron (Fe^2+^) succinate (B03AA06)	0.1% (122)	0.1% (167)	0.1% (161)	0.1% (146)	0.1% (134)	0.1% (100)
Iron (Fe^2+^) sulfate (B03AA07)	6.6% (8,550)	6.2% (8,168)	6.0% (7,865)	6.2% (8,369)	5.9% (7,619)	5.7% (7,419)
Iron trivalent, oral preparations (B03AB)	**0.2% ** **(296)**	**0.5% ** **(648)**	**0.6% (829)**	**0.6% (767)**	**0.6% (824)**	**0.7% (896)**
Ferric oxide polymaltose complexes (B03AB05)	0.2% (293)	0.4% (575)	0.5% (651)	0.3% (467)	0.3% (362)	0.2% (303)
Ferric maltol (B03AB10)	- (< 5)	0.1% (73)	0.1% (178)	0.2% (300)	0.4% (462)	0.5% (593)
Iron, parenteral preparations (B03AC)	**5.8% (7**,**489)**	**6.1% (8**,**061)**	**6.2% (8**,**158)**	**6.5% (8**,**798)**	**6.3% (8**,**115)**	**6.5% (8**,**445)**
Ferric oxide polymaltose complexes (B03AC01)	2.9% (3,672)	3.2% (4,247)	3.4% (4,471)	3.5% (4,709)	3.4% (4,417)	3.9% (5,067)
Saccharated iron oxide (B03AC02)	0.6% (749)	0.6% (769)	0.5% (723)	0.6% (780)	0.6% (739)	0.6% (742)
Ferric oxide dextran complex (B03AC06)	0.0% (43)	0.0% (42)	0.0% (32)	0.0% (45)	0.0% (26)	0.0% (17)
Ferric sodium gluconate complex (B03AC07)	2.4% (3,137)	2.4% (3,124)	2.3% (3,053)	2.2% (2,995)	2.0% (2,643)	1.9% (2,498)
Ferric derisomaltose (B03AC08)	0.1% (111)	0.1% (144)	0.1% (158)	0.6% (756)	0.6% (724)	0.3% (393)

*ATC* Anatomical Therapeutic Chemical, *ID/IDA* iron deficiency/iron deficiency anemia

For the rarely prescribed oral trivalent iron preparations, however, the use of ferric maltol increased slightly between 2018 and 2021 when compared to ferric oxide polymaltose complexes (Fig. 4B). Data on other subpopulations of patients with ID/IDA are provided in Additional file 1, Supplementary Tables 6–10.

### Healthcare resource utilization and expenditures

In the population of patients with ID/IDA, the percentages of both hospitalized individuals (all-cause) and individuals on sick leave were higher than in the matched control group (41.2% vs. 22.0%; 21.4% vs. 3.1%). These differences corresponded to higher mean total costs (all-cause) in patients with ID/IDA than in the matched control group (Table 4).

**Table 4:** Healthcare resource utilization in patients with ID/IDA and matched control individuals in Germany, 2021

	Population of patients with ID/IDA	Matched control population
Hospitalizations (all-cause)		
Patients ≥ 1 hospitalization, % (n)	41.2% (53,337)	22.0% (85,340)
Admissions per patient, mean ± SD	2.18 ± 1.77	1.80 ± 1.36
Length of stay (days), mean ± SD	23.74 ± 40.62	16.56 ± 37.76
Mean costs per individual ± SD	3,906 ± 11,877 €	1,112 ± 4,756 €
Hospitalizations (ID/IDA)		
Patients ≥ 1 hospitalization, % (n)	1.6% (2,023)	0.0% (0)
Admissions per patient, mean ± SD	1.08 ± 0.43	n.a.
Length of stay (days), mean ± SD	7.25 ± 8.38	n.a.
Mean costs per individual ± SD	52 ± 514 €	n.a.
Outpatient services (all-cause)		
Patients ≥ 1 utilization, % (n)	99.9% (129,372)	96.3% (374,127)
Number per patient, mean ± SD	29.91 ± 27.87	19.47 ± 17.31
Mean costs per individual ± SD	1,390 ± 3,119 €	795 ± 1,399 €
Outpatient services (ID/IDA)		
Patients ≥ 1 utilization, % (n)	91.1% (117,906)	0.0% (0)
Number per patient, mean ± SD	8.96 ± 15.87	n.a.
Mean costs per individual ± SD	395 ± 2,227 €	n.a.
Prescription drugs (any)		
Patients with ≥ 1 drug dispensation	92.0% (119,074)	80.3% (311,911)
Mean costs per individual ± SD	1,829 ± 16,979 €	778 ± 5,199 €
Prescription drug (iron preparations)		
Patients with ≥ 1 drug dispensation	30,9% (39,972)	1.0% (3,999)
Mean costs per individual ± SD	25 ± 101 €	1 ± 14 €
Total costs* per individual		
All cause mean costs ± SD	7,125 ± 21,867 €	2,685 ± 7,820 €
ID/IDA-related mean costs ± SD	472 ± 2,303 €	1 ± 14 €
Sick leaves		
Patients with valid data, n	129,362	388,022
Sick leaves (all-cause)		
Patients ≥ 1 sick leave, % (n)	21.4% (27,689)	3.1% (11,882)
Number per patient, mean ± SD	2.29 ± 2.03	1.77 ± 1.58
Length (days), mean ± SD	40.76 ± 90.88	64.54 ± 98.11
Sick leave (due to ID/IDA)		
Patients ≥ 1 sick leave, % (n)	0.4% (462)	0.0% (0)
Number per patient, mean ± SD	1.14 ± 1.33	n.a.
Length (days), mean ± SD	21.31 ± 59.96	n.a.

*ID/IDA* iron deficiency/iron deficiency anemia, *SD* standard deviation

*Total costs included costs due to hospitalizations, outpatient services, and drug prescriptions

ID/IDA-related costs (mean ± SD) for patients with ID/IDA (472 ± 2,303 €) further increased with concurrent diseases and were highest for patients with concurrent heart disease (853 ± 3,535 €). HRU and associated expenditures in subgroups are depicted in Additional file 1, Supplementary Tables 11–12.

#### Ferric maltol treatment

One year after treatment initiation with ferric maltol, HRU of the treatment cohort was lower in important parameters when compared to the one-year period before the index date (Table 5).

**Table 5:** Healthcare resource utilization in patients with ID/IDA treated with ferric maltol (*n* = 686) before and after treatment initiation (index date)

	1 year before index	1 year after index (including the index date)
Hospitalizations (all-cause)		
Patients ≥ 1 hospitalization, % (n)	52.0% (357)	47.7% (327)
Admissions per patient, mean ± SD	2.61 ± 2.15	2.47 ± 1.83
Length of stay (days), mean ± SD	23.78 ± 36.20	26.53 ± 51.44
Mean costs per individual ± SD	5,000 ± 11,460 €	3,873 ± 9,987 €
Hospitalizations (ID/IDA)		
Patients ≥ 1 hospitalization, % (n)	3.1% (21)	1.5% (10)
Admissions per patient, mean ± SD	1.14 ± 0.36	1.10 ± 0.32
Length of stay (days), mean ± SD	5.05 ± 3.50	6.70 ± 6.22
Mean costs per individual ± SD	77 ± 484 €	44 ± 737 €
Outpatient services (all-cause)		
Patients ≥ 1 utilization, % (n)	100.0% (686)	99.4% (682)
Number per patient, mean ± SD	40.57 ± 25.39	37.08 ± 27.93
Mean costs per individual ± SD	1,398 ± 1,594 €	1,450 ± 2,285 €
Outpatient services (ID/IDA)		
Patients ≥ 1 utilization, % (n)	96.4% (661)	68.8% (472)
Number per patient, mean ± SD	8.46 ± 8.12	10.80 ± 12.33
Mean costs per individual ± SD	219 ± 245 €	280 ± 1,440 €
Prescription drugs (any)		
Patients ≥ 1 drug dispensation, % (n)	97.1% (666)	100% (686)
Mean costs per individual ± SD	5,467 ± 13,829 €	6,543 ± 11,117 €
Prescription drugs (iron preparations)		
Patients ≥ 1 drug dispensation, % (n)	36.0% (247)	100% (686)
Mean costs per individual ± SD	62 ± 183 €	285 ± 315 €
Total costs per individual		
All cause mean costs ± SD	11,866 ± 18,170 €	11,866 ± 15,840 €
ID/IDA-related mean costs ± SD	358 ± 597 €	609 ± 1,674 €
Sick leaves		
Patients with valid data, n	686	686
Sick leaves (all-cause)		
Patients ≥ 1 sick leave, % (n)	33.5% (230)	32.1% (220)
Number per patient, mean ± SD	2.89 ± 2.11	2.59 ± 1.89
Length (days), mean ± SD	78.71 ± 136.35	72.33 ± 129.26
Sick leave (due to ID/IDA)		
Patients ≥ 1 sick leave, % (n)	1.9% (13)	0.7% (5)
Number per patient, mean ± SD	1.23 ± (0.44)	1.00 ± 0.00
Length (days), mean ± SD	33.31 (70.58)	13.60 (12.44)

*ID/IDA* iron deficiency/iron deficiency anemia, *SD* standard deviation, *Q1, Q3* quartile 1 and 3

*Total costs included costs due to hospitalizations, outpatient services, and drug prescriptions

These parameters included the percentage of patients with hospitalizations due to ID/IDA (1.5% vs. 3.1%), the percentage of patients using outpatient services due to ID/IDA (68.8% vs. 96.4%), and the percentage of patients with at least one sick leave due to ID/IDA (0.7% vs. 1.9%). These results were consistent among all subgroups for HRU (see Additional file 1, Supplementary Tables 13, 15, 17, 19, and 21) and associated costs (see Additional file 1, Supplementary Tables 14, 16, 18, 20, and 22). The total ID/IDA-related mean costs were higher after treatment initiation when compared to the pre-index period (609 ± 1,674 € vs. 358 ± 597 €), potentially driven by higher costs for drug treatment. However, all-cause costs remained approximately the same from the pre-index to the post-index period (11,866 ± 18,170 € vs. 11,866 ± 15,840 €; Table 5). It is interesting to note that the total mean treatment costs of this cohort were considerably higher than in the whole population of patients with ID/IDA, even before treatment initiation with ferric maltol (Tables 4 and 5).

Within the first 180 days after treatment initiation with ferric maltol, the majority of patients with ID/IDA and patients with ID/IDA with concurrent disease did not switch to any other iron preparation (Table 6).

**Table 6:** Patients switching from ferric maltol therapy to another iron formulation within 180 days after treatment initiation

	Treatment cohort and subgroups			
	ID/IDA % (n)	Heart disease % (n)	Inflamm. bowel disease % (n)	Menorrhagia& gynecol. diseases % (n)
Switch to parenteral iron prep.	16.8% (115)	13.0% (30)	22.7% (69)	14.9% (32)
Switch to bivalent oral iron prep.	6.1% (42)	10.4% (24)	2.6% (8)	4.7% (10)
Switch to ferric oxide polymaltose complexes	0% (0)	0% (0)	0% (0)	0% (0)
Switch to any iron prep.	22.9% (157)	23.4% (54)	25.3% (77)	19.5% (42)
No switch	77.1% (529)	76.6% (177)	74.7% (227)	80.5% (173)

*ID/IDA* iron deficiency/iron deficiency anemia

The post- vs. pre-index comparison revealed that ferric maltol treatment was associated with a considerable 29.0% point decrease in the proportion of patients receiving no treatment (from 64.0% before to 35.0% post index date; Table 7).

**Table 7:** Treatments of patients with ID/IDA of the ferric maltol treatment cohort during the year before and after treatment initiation at index date

Treatment option	ID/IDA (*n* = 686)	
	1 year before index *n* (%)	1 year after index *n* (%)
No treatment with iron preparations	439 (64.0%)	240 (35.0%)
Any treatment with iron preparations	247 (36.0%)	446 (65.0%)
thereof, treatment with bivalent oral iron preparations	141 (57.1%)	67 (15.0%)
thereof, treatment with ferric maltol	0 (0.0%)	304 (68.2%)
thereof, treatment with parenteral iron preparations	130 (52.6%)	167 (37.4%)

No patients received trivalent ferric oxide polymaltose in the year before and after the index date

*ID/IDA iron deficiency/iron deficiency anemia*

Among patients who received iron preparations, the proportion of those with parenteral iron preparations decreased (from 52.6% before to 37.4% post index date; Table 7). In the subgroups analyzed, the decrease in the proportion of patients receiving no treatment was between 26.5 (menorrhagia and other gynecological diseases; from 63.7% before to 37.2% post index date) to 33.3 (heart disease; from 63.6% before to 30.3% post index date) percentage points (see Additional file 1, Supplementary Tables 23–27).

## Discussion

Here, we report prevalence, associated comorbidities, and treatment patterns of patients with ID/IDA in Germany, 2016 to 2021. Our retrospective claim analysis revealed that overall the prevalence of ID/IDA was constant over the observational period at 3.34%. In women, prevalence was generally higher than in men, peaked at their reproductive age, and then increased from the age of 70. In men, prevalence increased with age. The number of comorbidities was higher in patients with ID/IDA than in age- and sex-matched control individuals and further increased with the concomitant diseases analyzed, namely heart disease, inflammatory bowel disease, and menorrhagia. Accordingly, ID/IDA was accompanied by elevated HRU and expenditures. ID/IDA-related costs increased with concurrent diseases and were highest for patients with ID/IDA with concurrent heart disease. Most patients with ID/IDA treated with iron supplementation received oral bivalent iron preparations, primarily iron sulfate, and parenteral iron supplementation. Oral trivalent iron preparations were rarely prescribed, but ferric maltol prescriptions increased during the study. This may be due to a better tolerance, as indicated by the low switching rates of patients with ID/IDA treated with ferric maltol.

The estimated prevalence of ID/IDA in Germany, as obtained by our retrospective claim analyses, was comparatively lower than in other western countries [30, 31], which might result from differences in the underlying data sets. The increased prevalence in women, however, is a well-known finding [3, 30, 32]. The etiology of ID/IDA in women is multifactorial, including menstrual blood loss, abnormal uterine bleeding, dietary lack or malabsorption of iron, as well as food intolerances [33]. Single factors such as heavy blood loss from menstruation or moderate blood loss in combination with other factors can lead to depletion of iron stores, resulting in ID/IDA [34, 35]. Maternal IDA is a risk factor for poor peri-operative, maternal, fetal, and neonatal outcomes [36]. Higher prevalence of ID/IDA was also observed in individuals with advanced age. Lower iron status and disturbed iron metabolism has been shown to be associated with several diseases and conditions related to old age, making an appropriate treatment mandatory to improve prognosis [37, 38].

When compared to matched control individuals, the comorbidity burden increased with ID/IDA and concurrent diseases, in particular heart disease. ID is often associated with chronic heart failure [39], and ID is diagnosed in up to 61.2% of heart failure patients with anemia and 45.6% without anemia [40]. Even in patients without anemia, ID worsens prognosis in heart failure. In inflammatory bowel disease and menorrhagia, ID/IDA has been shown to impair quality of life as well as physical and cognitive performance [41, 42]. Accordingly, HRU and associated expenditures were higher in patients with ID/IDA than in matched controls. ID/IDA-related costs increased with the presence of one of the concurrent diseases analyzed, with more all-cause hospitalizations, outpatient services, and sick-leaves per patient, as well as associated costs.

Once the underlying cause of ID/IDA has been identified and treated, an adequate iron supplementation therapy is required to correct hemoglobin levels and to refill iron stores. Practically, the oral route is considered as being the first-choice therapy, as this route allows the normal mechanism of absorption, presumably preventing the risk of iron overload. Oral iron is effective in many cases, cost-effective, and convenient as it is self-administered [43]. However, efficacy of oral iron may be limited by adverse effects such as gastrointestinal complaints reducing compliance [44]. A significant relationship was found between gastrointestinal side effects and non-adherence to therapy [45], and non-adherence has been estimated to range from 10 to 30% [46–48].

Bivalent iron preparations have been predominantly prescribed over trivalent iron preparations for many years [13]. In our study, most patients with ID/IDA who were prescribed iron preparations received at least one dispensation with an oral bivalent iron preparation, with the second most commonly prescribed treatment being parenteral. Oral trivalent iron preparations were prescribed very rarely. Within the group of oral bivalent iron preparations, ferrous sulphate preparations were most widely used.

A slight increase in use between 2018 and 2021 was observed for ferric maltol, a non-salt-based trivalent iron preparation, with improved tolerability in patients with ID/IDA with previous intolerance to other preparations. Indeed, most patients in our study did not switch from ferric maltol to another iron preparation during the follow-up, indicating good tolerability. In addition, among those treated with iron preparations, the proportion of patients receiving parenteral iron was lower in the year following initiation of treatment with ferric maltol. Growing familiarity of prescribers with the product may have also contributed to the increased use of ferric maltol, alongside its demonstrated tolerability and efficacy. Ferric maltol (brand name FERACCRU^®^) has been demonstrated to provide clinically meaningful hemoglobin improvements and a favorable safety profile, as demonstrated by phase-3 clinical trials [14, 49] and real-world evidence [50]. In patients with inflammatory bowel disease and ID/IDA, ferric maltol has been suggested as an alternative to parenteral iron supplementation [14]. Parenteral iron supplementation is the preferred treatment of ID/IDA in a variety of clinical situations. However, occasionally occurring adverse events following intravenous iron administration such as headache, nausea, hypo- and hypertension, rash, abdominal pain, and pruritus as well as rare but clinically more problematic conditions such as hypersensitivity reactions have prompted the search for alternative treatments [51, 52]. If future studies show that ferric maltol is better tolerated than oral bivalent and parenteral iron preparations, ferric maltol tolerability could have a positive effect on treatment adherence and outcomes.

In patients with ID/IDA treated with ferric maltol, ID/IDA-related costs slightly increased from the pre-index to the post-index period, whereas all-cause costs stayed virtually the same. Even before treatment initiation with ferric maltol, all-cause costs in the treatment cohort were considerably higher than in the overall population of patients with ID/IDA, including higher average costs for hospitalizations and drug treatment. According to treatment guidelines [10], trivalent iron preparations can be used as an alternative to bivalent iron in cases of intolerance or treatment failure. Thus, patients with more complex or severe disease, which are associated with higher healthcare utilization and costs, may be more likely to be treated with ferric maltol. However, further research is needed to understand the underlying drivers of these differences.

We recognized three major limitations in our study. First, only oral iron preparations prescribed by physicians and reimbursed by the SHI are classified as health claims and included in the database, whereas over-the-counter medications are not included even though many patients may buy their oral iron over the counter. Second, the InGef sample database employed in our study only contains data provided by selected German SHI companies. However, the representativeness for the German population in terms of sex, age, region, morbidity, mortality, and drug usage has been sufficiently demonstrated [17]. Third, our study did only include a post- vs. pre-index comparison of ferric maltol. The emphasis on ferric maltol in the study design was driven by funding from Norgine, market authorization holder of FERACCRU^®^ (ferric maltol). The authors affirm the funding did not influence study conduct, but this relationship should be considered when interpreting the findings. Comparative studies including comparisons with other iron preparations are needed to strengthen the argument for the relatively better tolerance of ferric maltol. Despite these limitations, this is the first study to examine the prevalence of ID/IDA, associated comorbidities, drug treatment patterns in such a large population in Germany.

## Conclusion

The prevalence of ID/IDA in Germany was constant over the observational period but was considerably higher in women of reproductive age and elderly people. Comorbidity burden was higher in patients with ID/IDA than in matched control individuals and further increased with the concomitant diseases analyzed, leading to elevated HRU and ID/IDA-related costs. Among patients with ID/IDA who were prescribed iron preparations, the majority received prescriptions for oral bivalent iron preparations or parenteral iron supplementation, while oral trivalent iron preparations were prescribed less frequently. Ferric maltol prescriptions increased, potentially because of a better tolerance, as indicated by low switching rates in patients with ID/IDA treated with ferric maltol.

In conclusion, continuous monitoring of the prevalence of ID/IDA might help to identify populations at increased health risk, such as women of reproductive age and the elderly, despite the heterogeneity of ID/IDA in terms of causes, comorbidities, and outcomes. We hope our analysis will help health specialists and policymakers to develop strategies to further reduce the burden of ID/IDA.

As such the authors are either direct employees of Norgine, the funder of this study, or have acted as consultants or sub-contractors on the project. Norgine is the rights holder of FERACCRU^®^ (ferric maltol), which was partly a focal point of this investigation. The design of the study, which focuses partly on Norgine’s product, has been influenced by funding from Norgine. While the authors assert that this funding did not impact the conduct of the study, the interpretation of the results, and the right to publish the results without limitation, the focus on ferric maltol reflects the interests of the funding source. The authors have not included comparative analyses of other iron deficiency treatments in this manuscript.

## Supplementary Information

Additional File 1.

## Data Availability

The data analyzed in this study was retrieved from the Institute for Applied Health Research Berlin (InGef) Research Database and cannot be made available in the manuscript, additional files, or in a public repository due to German data protection laws (Bundesdatenschutzgesetz).To facilitate the replication of results, anonymized data used for this study are stored on a secure drive at the InGef GmbH. Access to the data used in this study can only be provided to external parties under the conditions of the cooperation contract of this research project and can be assessed upon request, after written approval (contact: info@ingef.de), if required.

## Abbreviations

ATC: Anatomical Therapeutic Chemical
EBM: ‘Einheitlicher Bewertungsmaßstab’ (doctor’s fee scale)
ECI: Elixhauser Comorbidity Index
HRU: Healthcare resource utilization
IBD: Inflammatory bowel disease
ICD-10-GM: International Classification of Diseases, 10th version, German modification
ID: Iron deficiency
IDA: Iron deficiency anemia
OPS: ‘Operationen- und Prozedurenschlüssel’ (German modification of the International Classification of Procedures in Medicine)
Q1, Q3: quartile 1 and 3, interquartile range
SD: Standard deviation
SHI: Statutory health insurance
