# Supplementary material for: Epidemiology and treatment of patients with iron deficiency and iron deficiency anemia: a study on longitudinal German health claims data
Source: BMC Public Health. 2025 Sep 24;25:3053. doi: 10.1186/s12889-025-24730-9 (PMC12459059; doi:10.1186/s12889-025-24730-9)
Supplement: Supplementary file 1 — Additional File 1. [file 12889_2025_24730_MOESM1_ESM.docx]

# Additional file 1

## Supplementary Table 1

Operationalization of pre-defined diagnoses

| **Diagnosis** | **Coding** | **Description** |
| --- | --- | --- |
| Type 2 diabetes mellitus | ICD-10-GM: E11.- | Type 2 diabetes mellitus |
| Unspecified diabetes mellitus | ICD-10-GM: E14.- | Unspecified diabetes mellitus |
| Chronic ischemic heart disease | ICD-10-GM: I25.- | Chronic ischemic heart disease |
| Nonrheumatic mitral valve disorders | ICD-10-GM: I34.- | Nonrheumatic mitral valve disorders |
| Nonrheumatic aortic valve disorders | ICD-10-GM: I35.- | Nonrheumatic aortic valve disorders |
| Atrial fibrillation and flutter | ICD-10-GM: I48.- | Atrial fibrillation and flutter |
| Other cardiac arrhythmias | ICD-10-GM: I49.- | Other cardiac arrhythmias |
| Heart failure | ICD-10-GM: I50.- | Heart failure |
| Crohn’s disease | ICD-10-GM: K50.- | Crohn’s disease |
| Colitis ulcerosa | ICD-10-GM: K51.- | Colitis ulcerosa |
| Other noninfective gastroenteritis and colitis | ICD-10-GM: K52.- | Other noninfective gastroenteritis and colitis |
| Gonarthrosis | ICD-10-GM: M17.- | Gonarthrosis [arthrosis of knee] |
| Other arthrosis | ICD-10-GM: M19.- | Other arthrosis |
| Other non-inflamm. disorders of uterus, except cervix | ICD-10-GM: N85.- | Other noninflammatory disorders of uterus, except cervix |
| Absent, scanty and rare menstruation | ICD-10-GM: N91.- | Absent, scanty and rare menstruation |
| Excessive, frequent and irregular menstruation | ICD-10-GM: N92.- | Excessive, frequent and irregular menstruation |
| Other abnormal uterine and vaginal bleeding | ICD-10-GM: N93.- | Other abnormal uterine and vaginal bleeding |
| Anemia complicating pregnancy, childbirth and the puerperium | ICD-10-GM: O99.0 | Anemia complicating pregnancy, childbirth and the puerperium |
| Pregnancy* | EBM: 01770 | Care of a pregnant woman |
|  | EBM: 32007 | Maternity care services (maternity guidelines of the G-BA for substitution, emergency or co- or further treatment) |
|  | EBM: 01904 | Abortion, medical or criminological indication, up to the 12^th^ (p.c.) or 14^th^ (p.m.) completed week of pregnancy |
|  | EBM: 01905 | Abortion, medical indication, up to the 13^th^ (p.c.) or 15^th^ (p.m.) week of pregnancy |
|  | EBM: 01906 | Drug induced abortion, medical or criminological indication, up to the 49^th^ day (p.m.) |
|  | EBM: 01913 | Anesthesia in the context of an abortion |
|  | EBM: 01914 | Monitoring and care of the patient after an abortion following service according to no. 01913 |
|  | EBM: 01784 | Amnioscopy |
|  | EBM: 01815 | Examination, counselling for women in childbed (sections F.1 or F.2 of the maternity guidelines) |
|  | EBM: 08410 | Stay in the delivery room without rendering billable services due to delivery care, per completed 30 minutes |
|  | EBM: 08411 | Delivery care and supervision |
|  | EBM: 08412 | Surcharge in addition to service (no. 08411) for care and delivery during a complicated birth |
|  | EBM: 08413 | External cephalic version |
|  | EBM: 08414 | Internal cephalic or combined version |
|  | EBM: 08415 | Surcharge in addition to service (no. 08411) for care and delivery for birth by Caesarean section |
|  | EBM: 08416 | Surcharge in addition to service (no. 08411) for the removal of the placenta |
|  | ICD-10-GM: O00.- | Ectopic pregnancy |
|  | ICD-10-GM: O01.- | Hydatidiform mole |
|  | ICD-10-GM: O02.- | Other abnormal products of conception |
|  | ICD-10-GM: O03.- | Spontaneous abortion |
|  | ICD-10-GM: O04.- | Medical abortion |
|  | ICD-10-GM: O05.- | Other abortion |
|  | ICD-10-GM: O06.- | Unspecified abortion |
|  | ICD-10-GM: O07.- | Failed attempted abortion |
|  | ICD-10-GM: O08.- | Complications following abortion and ectopic and molar pregnancy |
|  | ICD-10-GM: O14.- | Pre-eclampsia |
|  | ICD-10-GM: O15.- | Eclampsia |
|  | ICD-10-GM: O48.- | Prolonged pregnancy |
|  | ICD-10-GM: O60.- | Preterm labor and delivery |
|  | ICD-10-GM: O61.- | Failed induction of labor |
|  | ICD-10-GM: O62.- | Abnormalities of forces of labor |
|  | ICD-10-GM: O63.- | Long labor |
|  | ICD-10-GM: O64.- | Obstructed labor due to malposition and malpresentation of fetus |
|  | ICD-10-GM: O65.- | Obstructed labor due to maternal pelvic abnormality |
|  | ICD-10-GM: O66.- | Other obstructed labor |
|  | ICD-10-GM: O67.- | Labor and delivery complicated by intrapartum hemorrhage, not elsewhere classified |
|  | ICD-10-GM: O68.- | Labor and delivery complicated by fetal stress [distress] |
|  | ICD-10-GM: O69.- | Labor and delivery complicated by umbilical cord complications |
|  | ICD-10-GM: O70.- | Perineal laceration during delivery |
|  | ICD-10-GM: O71.- | Other obstetric trauma |
|  | ICD-10-GM: O72.- | Postpartum hemorrhage |
|  | ICD-10-GM: O73.- | Retained placenta and membranes, without hemorrhage |
|  | ICD-10-GM: O74.- | Complications of anesthesia during labor and delivery |
|  | ICD-10-GM: O75.- | Other complications of labor and delivery, not elsewhere classified |
|  | ICD-10-GM: O80.- | Single spontaneous delivery |
|  | ICD-10-GM: O81.- | Single delivery by forceps or vacuum extractor |
|  | ICD-10-GM: O82.- | Single delivery by caesarean section |
|  | ICD-10-GM: O85.- | Puerperal sepsis |
|  | ICD-10-GM: O86.- | Other puerperal infections |
|  | ICD-10-GM: O87.- | Venous complications and hemorrhoids in the puerperium |
|  | ICD-10-GM: O88.- | Obstetric embolism |
|  | ICD-10-GM: O89.- | Complications of anesthesia during the puerperium |
|  | ICD-10-GM: O90.- | Complications of the puerperium, not elsewhere classified |
|  | ICD-10-GM: O99.0 | Anemia complicating pregnancy, childbirth and the puerperium |
|  | ICD-10-GM: P05.- | Slow fetal growth and fetal malnutrition |
|  | ICD-10-GM: P07.- | Disorders related to short gestation and low birth weight, not elsewhere classified |
|  | ICD-10-GM: P08.- | Disorders related to long gestation and high birth weight |
|  | ICD-10-GM: P95.- | Fetal death of unspecified cause |
|  | ICD-10-GM: Z33.- | Pregnant state, incidental |
|  | ICD-10-GM: Z34.- | Supervision of normal pregnancy |
|  | ICD-10-GM: Z35.- | Supervision of high-risk pregnancy |
|  | ICD-10-GM: Z36.- | Antenatal screening |
|  | ICD-10-GM: Z37.- | Outcome of delivery |
|  | ICD-10-GM: Z38.- | Liveborn infants according to place of birth |
|  | ICD-10-GM: Z39.- | Postpartum care and examination |
|  | OPS: 5-720 | Breech delivery / instrumental delivery: Forceps delivery |
|  | OPS: 5-724 | Breech delivery/ instrumental delivery: Rotation of the baby’s head with forceps |
|  | OPS: 5-725 | Breech delivery / instrumental delivery: Breech extraction |
|  | OPS: 5-727 | Breech delivery / instrumental delivery: Spontaneous and vaginal surgical breech delivery |
|  | OPS: 5-728 | Breech delivery / instrumental delivery: Vacuum delivery |
|  | OPS: 5-729 | Breech delivery / instrumental delivery: Other instrumental delivery |
|  | OPS: 5-730 | Other operations for induction of labor and during birth: Artificial rupture of amniotic sac [amniotomy] |
|  | OPS: 5-731 | Other operations for induction of labor and during birth: Other surgical induction of labor |
|  | OPS: 5-732 | Other operations for induction of labor and during birth: Internal and combined rotation without and with extraction |
|  | OPS: 5-733 | Other operations for induction of labor and during birth: Failed vaginal surgical delivery |
|  | OPS: 5-734 | Other operations for induction of labor and during birth: Surgical measures on the fetus to facilitate birth |
|  | OPS: 5-738 | Other operations for induction of labor and during birth: Episiotomy and suture |
|  | OPS: 5-739 | Other operations for induction of labor and during birth: Other operations to assist the birth |
|  | OPS: 5-740 | Caesarean section and child development: Classic caesarean section |
|  | OPS: 5-741 | Caesarean section and child development: Caesarean section, supracervical and corporeal |
|  | OPS: 5-742 | Caesarean section and child development: Extraperitoneal cesarean section |
|  | OPS: 5-743 | Caesarean section and child development: Removal of an intraperitoneal embryo |
|  | OPS: 5-744 | Caesarean section and child development: Operations in extrauterine pregnancy |
|  | OPS: 5-749 | Caesarean section and child development: Other caesarean section |
|  | OPS: 5-750 | Intraamnial injection to terminate the pregnancy |
|  | OPS: 5-751 | Curettage to terminate the pregnancy [abruptio] |
|  | OPS: 5-752 | Other operations to terminate a pregnancy |
|  | OPS: 5-753 | Therapeutic amniocentesis [puncture of amniotic sac] |
|  | OPS: 5-754 | Intrauterine therapy for the fetus |
|  | OPS: 5-755 | Other intrauterine operations on the fetus |
|  | OPS: 5-756 | Removal of retained placenta (postpartal) |
|  | OPS: 5-757 | Extirpation of uterus, to aid birth |
|  | OPS: 5-758 | Reconstruction of female genital organs after rupture, postpartum [perineal laceration] |
|  | OPS: 5-759 | Other birth-assisting operations |
|  | OPS: 1-699.2 | Amnioscopy |
|  | OPS: 9-280 | Treatment during the pregnancy: Inpatient treatment prior to birth during the same hospital admission |
|  | OPS: 9-260 | Perinatal measures: Monitoring and management of normal birth |
|  | OPS: 9-261 | Perinatal measures: Monitoring and management of high-risk birth |
|  | OPS: 9-262 | Perinatal measures: Postnatal care of newborn |
|  | OPS: 9-263 | Perinatal measures: Monitoring and management of birth of placenta in separate location |
|  | OPS: 9-268 | Perinatal measures: Monitoring and management of birth, unspecified |

EMB: Doctor's Fee Scale (‘Einheitlicher Bewertungsmaßstab’); ICD-10-GM: German modification of the 10th version of the International Classification of Diseases; OPS: German modification of the International Classification of Procedures in Medicine (‘Operationen- und Prozedurenschlüssel’); p.c.: post conceptionem; p.m.: post menstruationem; *Documentation of one of the listed codes during the respective calendar year is sufficient to meet the identification criterium for ‘pregnancy’

## Supplementary Table 2

Selection steps for populations and subpopulations of patients with ID/IDA, 2016 – 2021

| **Selection step** | **Year** | | | | | |
| --- | --- | --- | --- | --- | --- | --- |
|  | **2016** | **2017** | **2018** | **2019** | **2020** | **2021** |
| *Number of individuals in the InGef sample database* | | | | | | |
|  | 4,162,138 | 4,191,503 | 4,271,158 | 4,278,748 | 4,278,800 | 4,345,882 |
| *Thereof, number of individuals who were continuously insured or continuously insured until death* | | | | | | |
|  | 4,037,704 | 4,048,286 | 4,011,147 | 4,028,026 | 4,010,693 | 3,876,820 |
| *Thereof, number of individuals with prevalent ID or IDA* | | | | | | |
|  | 128,746 | 132,420 | 132,179 | 135,444 | 129,716 | 129,462 |
| *Thereof, met the inclusion criteria for the following subpopulation:* | | | | | | |
| - Heart disease | 41,148 | 43,197 | 44,450 | 46,571 | 44,699 | 44,535 |
| - Geriatric | 36,547 | 38,620 | 39,785 | 41,642 | 40,589 | 40,662 |
| - Menorrhagia or other gynecological diseases | 37,362 | 37,667 | 37,091 | 37,436 | 34,652 | 34,421 |
| - Gastrointestinal bleeding | 21,277 | 21,766 | 22,200 | 22,795 | 20,838 | 20,417 |
| - Inflammatory bowel disease | 10,603 | 10,350 | 10,426 | 10,397 | 8,724 | 8,422 |

ID/IDA: iron deficiency/iron deficiency anemia

## Supplementary Table 3

Selection steps for the ferric maltol treatment cohort and subcohorts

| Selection step | Index period  (2017 – 2020) |
| --- | --- |
| *Number of individuals within the population of patients with ID/IDA during the index period.* | |
|  | 376,139 |
| *Thereof, ≥ 1 dispensation with ferric maltol in the quarter of ID/IDA diagnosis during the index period; observational data available at least 365 days before and 365 days after the dispensation.* | |
|  | **747** |
| *Thereof, had no dispensation with ferric maltol in the 365 days prior to index date (=treatment cohort).* | |
|  | **686** |
| *Thereof, met the inclusion criteria for the following subcohorts.* | |
| Heart disease  Geriatric  Menorrhagia or other gynecological diseases  Gastrointestinal bleeding  Inflammatory bowel disease | 231  168  215  377  304 |

ID/IDA: iron deficiency/iron deficiency anemia

## Supplementary Table 4

Comorbidity burden in patients with ID/IDA, matched controls, and subpopulations (Mean number of diseases of the Elixhauser Comorbidity Index ± SD).

| **Population** | **2016** | **2017** | **2018** | **2019** | **2020** | **2021** |
| --- | --- | --- | --- | --- | --- | --- |
| Patients with ID/IDA | 4.26  ± 3.46 | 4.34  ± 3.52 | 4.46  ± 3.57 | 4.52  ± 3.60 | 4.55  ± 3.61 | 4.53  ± 3.61 |
| Matched control population | 2.12 ±2.44 | 2.18  ± 2.49 | 2.23  ± 2.52 | 2.27  ± 2.56 | 2.25  ± 2.54 | 2.27  ± 2.53 |
| Heart disease | 7.60  ± 3.50 | 7.73  ± 3.49 | 7.83  ± 3.49 | 7.86  ± 3.50 | 7.89  ± 3.51 | 7.88  ± 3.50 |
| Geriatric | 7.54  ± 3.44 | 7.63  ± 3.44 | 7.72  ± 3.46 | 7.78  ± 3.46 | 7.79  ± 3.47 | 7.76  ± 3.45 |
| Menorrhagia or other gynecological diseases | 3.29  ± 2.63 | 3.33  ± 2.66 | 3.40  ± 2.72 | 3.43  ± 2.75 | 3.46  ± 2.76 | 3.44  ± 2.75 |
| Gastrointestinal bleeding | 6.03  ± 4.04 | 6.21  ± 4.06 | 6.30  ± 4.08 | 6.39  ±4.10 | 6.59  ± 4.09 | 6.64  ± 4.05 |
| Inflammatory bowel disease | 4.52  ± 3.72 | 4.68  ± 3.80 | 4.69  ± 3.82 | 4.72  ± 3.80 | 4.97  ± 3.89 | 6.64  ± 4.05 |

ID/IDA: iron deficiency/iron deficiency anemia; SD: standard deviation

## Supplementary Table 5

Prevalence of pre-defined diagnoses in patients with ID/IDA of advanced age (≥70; geriatric subpopulation) and with concurrent gastrointestinal bleeding in Germany, 2021

| **Diagnosis** | **Prevalence (n)** |
| --- | --- |
| **Subpopulation: Geriatric** |  |
| Type 2 diabetes mellitus | 43.6% (17,721) |
| Heart failure | 41.6% (16,901) |
| Chronic ischaemic heart disease | 40.8% (16,580) |
| Atrial fibrillation and flutter | 36.0% (14,643) |
| Gonarthrosis [arthrosis of knee] | 29.2% (11,854) |
| Unspecified diabetes mellitus | 22.5% (9,163) |
| Nonrheumatic mitral valve disorders | 19.9% (8,079) |
| Coxarthrosis [arthrosis of hip] | 19.4% (7,884) |
| Nonrheumatic aortic valve disorders | 18.2% (7,412) |
| Other arthrosis | 18.2% (7,387) |
| **Subpopulation: Gastrointestinal bleeding** |  |
| Type 2 diabetes mellitus | 31.0% (6,326) |
| Heart failure | 27.2% (5,559) |
| Chronic ischaemic heart disease | 27.2% (5,552) |
| Other noninfective gastroenteritis and colitis | 25.2% (5,147) |
| Atrial fibrillation and flutter | 23.2% (4,742) |
| Polyp of colon | 19.8% (4,038) |
| Gonarthrosis [arthrosis of knee] | 19.7% (4,031) |
| Benign neoplasm of colon, rectum, anus and anal canal | 16.8% (3,432) |
| Unspecified diabetes mellitus | 15.8% (3,227) |
| Nonrheumatic mitral valve disorders | 14.0% (2,866) |

## Supplementary Table 6

Dispensations of supplemental iron preparations in Germany, 2016 – 2021 (Subgroup: Heart disease)

|  | **Percentage of patients with ID/IDA (n)** | | | | | |
| --- | --- | --- | --- | --- | --- | --- |
| **Class / agent (ATC-Code)** | **2016**  **(41,148)** | **2017**  **(43,197)** | **2018**  **(44,450)** | **2019**  **(46,571)** | **2020**  **(44,699)** | **2021**  **(44,535)** |
| **Iron bivalent, oral preparations (B03AA)** | **32.8% (13,499)** | **32.0% (13,822)** | **31.4% (13,953)** | **31.5% (14,680)** | **30.7% (13,723)** | **30.2% (13,448)** |
| Iron (Fe^2+^) glycine sulfate  (B03AA01) | 26.5% (10,924) | 26.1% (11,284) | 25.8% (11,460) | 25.7% (11,973) | 25.2% (11,242) | 24.9% (11,103) |
| Ferrous fumarate  (B03AA02) | 0.1% (59) | 0.1% (60) | 0.1% (55) | 0.1% (54) | 0.1% (45) | 0.1% (53) |
| Iron (Fe^2+^) gluconate  (B03AA03) | 0.5% (224) | 0.6% (245) | 0.5% (201) | 0.4% (168) | 0.3% (141) | 0.3% (142) |
| Iron (Fe^2+^) succinate  (B03AA06) | 0.1% (50) | 0.2% (71) | 0.1% (65) | 0.1% (63) | 0.1% (66) | 0.1% (29) |
| Iron (Fe^2+^) sulfate  (B03AA07) | 6.4% (2,647) | 5.8% (2,500) | 5.7% (2,514) | 6.0% (2,777) | 5.7% (2,547) | 5.5% (2,443) |
| **Iron trivalent, oral preparations (B03AB)** | **0.1% (21)** | **0.1% (57)** | **0.2% (81)** | **0.2% (104)** | **0.4% (199)** | **0.5% (226)** |
| Ferric oxide polymaltose complexes (B03AB05) | 0.1% (21) | 0.1% (47) | 0.1% (37) | 0.1% (27) | 0.1% (37) | 0.0% (19) |
| Ferric maltol  (B03AB10) | 0.0%  (0) | 0.0% (10) | 0.1% (44) | 0.2% (77) | 0.4% (162) | 0.5% (207) |
| **Iron, parenteral preparations (B03AC)** | **7.2% (2,948)** | **7.5% (3,240)** | **7.5% (3,348)** | **7.8% (3,625)** | **7.6% (3,415)** | **8.0% (3,558)** |
| Ferric oxide polymaltose complexes (B03AC01) | 3.2% (1,324) | 3.7% (1,601) | 3.8% (1,706) | 3.9% (1,814) | 3.9% (1,738) | 4.5% (2,001) |
| Saccharated iron oxide  (B03AC02) | 1.0% (403) | 1.0% (431) | 1.0% (432) | 1.1% (491) | 1.0% (468) | 1.1% (472) |
| Ferric oxide dextran complex (B03AC06) | 0.1% (25) | 0.1% (26) | 0.0% (15) | 0.1% (26) | 0.0% (14) | 0.0% (11) |
| Ferric sodium gluconate complex (B03AC07) | 3.0% (1,250) | 2.9% (1,257) | 2.9% (1,289) | 2.7% (1,235) | 2.5% (1,121) | 2.4% (1,059) |
| Ferric derisomaltose  (B03AC08) | 0.1% (43) | 0.2% (67) | 0.1% (63) | 0.6% (290) | 0.7% (294) | 0.4% (163) |

ATC: Anatomical Therapeutic Chemical; ID/IDA: iron deficiency/iron deficiency anemia

## Supplementary Table 7

Dispensations of supplemental iron preparations in Germany, 2016 – 2021 (Subgroup: Geriatric)

|  | **Percentage of patients with ID/IDA (n)** | | | | | |
| --- | --- | --- | --- | --- | --- | --- |
| **Class / agent (ATC-Code)** | **2016**  **(36,547)** | **2017**  **(38,620)** | **2018**  **(39,785)** | **2019**  **(41,642)** | **2020**  **(40,589)** | **2021**  **(40,662)** |
| **Iron bivalent, oral preparations (B03AA)** | **35.1% (12,818)** | **34.6% (13,347)** | **33.9% (13,474)** | **33.9% (14,127)** | **33.5% (13,589)** | **32.5% (13,208)** |
| Iron (Fe^2+^) glycine sulfate  (B03AA01) | 28.4% (10,370) | 28.4% (10,951) | 28.0% (11,128) | 27.9% (11,608) | 27.6% (11,197) | 27.1% (11,013) |
| Ferrous fumarate  (B03AA02) | 0.1% (53) | 0.1% (56) | 0.1% (47) | 0.1% (41) | 0.1% (47) | 0.1% (50) |
| Iron (Fe^2+^) gluconate  (B03AA03) | 0.6% (226) | 0.6% (232) | 0.5% (189) | 0.4% (159) | 0.3% (126) | 0.3% (126) |
| Iron (Fe^2+^) succinate  (B03AA06) | 0.1% (46) | 0.2% (60) | 0.1% (52) | 0.2% (63) | 0.2% (61) | 0.1% (28) |
| Iron (Fe^2+^) sulfate  (B03AA07) | 6.8% (2,479) | 6.1% (2,348) | 5.9% (2,364) | 6.2% (2,569) | 6.1% (2,466) | 5.7% (2,324) |
| **Iron trivalent, oral preparations (B03AB)** | **0.0% (14)** | **0.1% (25)** | **0.1% (38)** | **0.2% (70)** | **0.4% (148)** | **0.5% (189)** |
| Ferric oxide polymaltose complexes (B03AB05) | 0.0% (14) | 0.1% (20) | 0.0% (11) | 0.0%  (7) | 0.0% (11) | 0.0%  (5) |
| Ferric maltol  (B03AB10) | 0.0%  (0) | 0.0%  (5) | 0.1% (27) | 0.2% (63) | 0.3% (137) | 0.5% (184) |
| **Iron, parenteral preparations (B03AC)** | **6.6% (2,415)** | **6.9% (2,682)** | **6.9% (2,737)** | **7.2% (2,996)** | **7.0% (2,829)** | **7.3% (2,973)** |
| Ferric oxide polymaltose complexes (B03AC01) | 3.0% (1,101) | 3.5% (1,368) | 3.6% (1,423) | 3.6% (1,499) | 3.7% (1,483) | 4.3% (1,735) |
| Saccharated iron oxide  (B03AC02) | 0.9% (325) | 0.8% (318) | 0.8% (328) | 0.9% (358) | 0.9% (352) | 0.9% (360) |
| Ferric oxide dextran complex (B03AC06) | 0.1% (22) | 0.1% (20) | 0.0%  (9) | 0.0% (15) | 0.0%  (6) | -%  (<5) |
| Ferric sodium gluconate complex (B03AC07) | 2.8% (1,015) | 2.7% (1,039) | 2.6% (1,028) | 2.5% (1,049) | 2.2% (908) | 2.0% (833) |
| Ferric derisomaltose  (B03AC08) | 0.1% (24) | 0.1% (52) | 0.1% (59) | 0.6% (259) | 0.6% (255) | 0.4% (146) |

ATC: Anatomical Therapeutic Chemical; ID/IDA: iron deficiency/iron deficiency anemia

## Supplementary Table 8

Dispensations of iron preparations in Germany, 2016 – 2021 (Subgroup: menorrhagia or other gynecological diseases)

|  | **Percentage of patients with ID/IDA (n)** | | | | | |
| --- | --- | --- | --- | --- | --- | --- |
| **Class / agent (ATC-Code)** | **2016**  **(37,362)** | **2017**  **(37,667)** | **2018**  **(37,091)** | **2019**  **(37,436)** | **2020**  **(34,652)** | **2021**  **(34,421)** |
| **Iron bivalent, oral preparations (B03AA)** | **28.8% (10,759)** | **27.7% (10,429)** | **26.6% (9,884)** | **26.8% (10,031)** | **25.7% (8,906)** | **25.3% (8,699)** |
| Iron (Fe^2+^) glycine sulfate  (B03AA01) | 19.% (7,173) | 18.4% (6,930) | 17.9% (6,626) | 17.5% (6,565) | 17.1% (5,927) | 17.0% (5,853) |
| Ferrous fumarate  (B03AA02) | 0.1% (33) | 0.1% (51) | 0.1% (35) | 0.1% (38) | 0.1% (36) | 0.1% (38) |
| Iron (Fe^2+^) gluconate  (B03AA03) | 0.6% (209) | 0.6% (235) | 0.5% (176) | 0.5% (174) | 0.4% (155) | 0.4% (126) |
| Iron (Fe^2+^) succinate  (B03AA06) | 0.1% (29) | 0.1% (52) | 0.1% (45) | 0.1% (33) | 0.1% (39) | 0.1% (30) |
| Iron (Fe^2+^) sulfate  (B03AA07) | 9.8% (3,668) | 9.2% (3,484) | 8.9% (3,315) | 9.4% (3,512) | 8.7% (3,030) | 8.4% (2,893) |
| **Iron trivalent, oral preparations (B03AB)** | **0.0% (5)** | **0.1% (32)** | **0.2% (58)** | **0.2% (71)** | **0.4% (140)** | **0.5% (169)** |
| Ferric oxide polymaltose complexes (B03AB05) | -%  (<5) | 0.0% (16) | 0.0% (13) | 0.0% (6) | 0.0% (16) | 0.0% (7) |
| Ferric maltol  (B03AB10) | -%  (<5) | 0.0% (16) | 0.1% (45) | 0.2% (65) | 0.4% (124) | 0.5% (162) |
| **Iron, parenteral preparations (B03AC)** | **6.3% (2,360)** | **6.5% (2,449)** | **6.4% (2,364)** | **6.8% (2,551)** | **6.6% (2,280)** | **6.8% (2,343)** |
| Ferric oxide polymaltose complexes (B03AC01) | 3.2% (1,202) | 3.6% (1,358) | 3.7% (1,376) | 3.7% (1,384) | 3.7% (1,294) | 4.2% (1,445) |
| Saccharated iron oxide  (B03AC02) | 0.4% (163) | 0.4% (166) | 0.3% (114) | 0.4% (142) | 0.3% (119) | 0.4% (135) |
| Ferric oxide dextran complex (B03AC06) | -%  (<5) | 0.0% (10) | 0.0% (8) | 0.0% (12) | 0.0% (6) | -%  (<5) |
| Ferric sodium gluconate complex (B03AC07) | 2.8% (1,031) | 2.5% (945) | 2.4% (896) | 2.5% (931) | 2.3% (789) | 2.1% (722) |
| Ferric derisomaltose  (B03AC08) | 0.1% (32) | 0.1% (40) | 0.1% (49) | 0.6% (206) | 0.5% (189) | 0.3% (107) |

ATC: Anatomical Therapeutic Chemical; ID/IDA: iron deficiency/iron deficiency anemia

## Supplementary Table 9

Dispensations of supplemental iron preparations in Germany, 2016 – 2021 (Subgroup: Gastrointestinal bleeding)

|  | **Percentage of patients with ID/IDA (n)** | | | | | |
| --- | --- | --- | --- | --- | --- | --- |
| **Class / agent (ATC-Code)** | **2016**  **(21,277)** | **2017**  **(21,766)** | **2018**  **(22,200)** | **2019**  **(22,795)** | **2020**  **(20,838)** | **2021**  **(20,417)** |
| **Iron bivalent, oral preparations (B03AA)** | **31.33% (6,663)** | **30.7% (6,676)** | **29.8% (6,615)** | **29.22% (6,660)** | **28.4% (5,924)** | **28.5% (5,821)** |
| Iron (Fe^2+^) glycine sulfate  (B03AA01) | 25.0% (5,309) | 24.8% (5,395) | 24.3% (5,393) | 23.6% (5,388) | 23.3% (4,847) | 23.7% (4,848) |
| Ferrous fumarate  (B03AA02) | 0.1% (31) | 0.2% (33) | 0.1% (31) | 0.1% (25) | 0.1% (20) | 0.1% (26) |
| Iron (Fe^2+^) gluconate  (B03AA03) | 0.7% (141) | 0.7% (163) | 0.5% (105) | 0.4% (85) | 0.3% (56) | 0.3% (64) |
| Iron (Fe^2+^) succinate  (B03AA06) | 0.1% (27) | 0.1% (24) | 0.1% (27) | 0.1% (23) | 0.1% (23) | 0.1% (12) |
| Iron (Fe^2+^) sulfate  (B03AA07) | 6.4% (1,356) | 5.7% (1,244) | 5.6% (1,245) | 5.7% (1,296) | 5.4% (1,130) | 5.1% (1,048) |
| **Iron trivalent, oral preparations (B03AB)** | **0.2% (44)** | **0.6% (136)** | **0.8% (174)** | **1.0% (218)** | **1.0% (217)** | **1.2% (237)** |
| Ferric oxide polymaltose complexes (B03AB05) | 0.2% (41) | 0.3% (75) | 0.3% (58) | 0.2% (36) | 0.1% (22) | 0.1% (19) |
| Ferric maltol  (B03AB10) | -% (<5) | 0.3% (61) | 0.5% (116) | 0.8% (182) | 0.9% (195) | 1.1% (218) |
| **Iron, parenteral preparations (B03AC)** | **9.6% (2,036)** | **10.1% (2,197)** | **10.0% (2,216)** | **10.5% (2,386)** | **10.3% (2,151)** | **10.8% (2,200)** |
| Ferric oxide polymaltose complexes (B03AC01) | 5.3% (1,125) | 6.1% (1,333) | 6.2% (1,368) | 6.3% (1,426) | 6.3% (1,320) | 7.0% (1,439) |
| Saccharated iron oxide  (B03AC02) | 0.8% (171) | 0.9% (186) | 0.8% (185) | 0.9% (199) | 1.0% (198) | 0.9% (184) |
| Ferric oxide dextran complex (B03AC06) | 0.1% (13) | 0.1% (15) | 0.0% (10) | 0.1% (21) | -%  (<5) | -% (<5) |
| Ferric sodium gluconate complex (B03AC07) | 3.6% (760) | 3.2% (706) | 3.2% (707) | 3.0% (680) | 2.6% (546) | 2.6% (539) |
| Ferric derisomaltose  (B03AC08) | 0.2% (39) | 0.2% (52) | 0.2% (43) | 1.0% (226) | 1.1% (224) | 0.6% (128) |

ATC: Anatomical Therapeutic Chemical; ID/IDA: iron deficiency/iron deficiency anemia

## Supplementary Table 10

Dispensations of supplemental iron preparations in Germany, 2016 – 2021 (Subgroup: Inflammatory bowel disease)

|  | **Percentage of patients with ID/IDA (n)** | | | | | |
| --- | --- | --- | --- | --- | --- | --- |
| **Class / agent (ATC-Code)** | **2016**  **(10,603)** | **2017**  **(10,350)** | **2018**  **(10,426)** | **2019**  **(10,397)** | **2020**  **(8,724)** | **2021**  **(8,422)** |
| **Iron bivalent, oral preparations (B03AA)** | **26.3% (2,785)** | **25.9% (2,680)** | **23.8% (2,481)** | **23.5% (2,447)** | **23.0% (2,008)** | **22.7% (1,914)** |
| Iron (Fe^2+^) glycine sulfate  (B03AA01) | 20.3% (2,150) | 20.2% (2,087) | 18.9% (1,969) | 18.3% (1,904) | 18.4% (1,606) | 18.5% (1,559) |
| Ferrous fumarate  (B03AA02) | 0.2% (16) | 0.1% (14) | 0.1% (15) | 0.1% (11) | 0.1% (10) | 0.1%  (8) |
| Iron (Fe^2+^) gluconate  (B03AA03) | 0.7% (77) | 0.9% (98) | 0.5% (51) | 0.3% (34) | 0.3% (26) | 0.3% (26) |
| Iron (Fe^2+^) succinate  (B03AA06) | 0.1%  (9) | 0.1% (12) | 0.1% (14) | 0.1%  (9) | 0.1%  (8) | 0.1%  (5) |
| Iron (Fe^2+^) sulfate  (B03AA07) | 5.9% (626) | 5.4% (556) | 4.8% (498) | 5.2% (545) | 4.7% (412) | 4.5% (377) |
| **Iron trivalent, oral preparations (B03AB)** | **0.4% (39)** | **1.3% (130)** | **1.6% (166)** | **1.8% (186)** | **1.9% (167)** | **2.1% (177)** |
| Ferric oxide polymaltose complexes (B03AB05) | 0.3% (36) | 0.7% (72) | 0.5% (54) | 0.3% (34) | 0.2% (19) | 0.2% (15) |
| Ferric maltol  (B03AB10) | -% (<5) | 0.6% (58) | 1.1% (112) | 1.5% (152) | 1.7% (148) | 1.9% (162) |
| **Iron, parenteral preparations (B03AC)** | **10.7% (1,130)** | **11.0% (1,137)** | **11.1% (1,162)** | **11.6% (1,202)** | **12.7% (1,109)** | **13.3% (1,118)** |
| Ferric oxide polymaltose complexes (B03AC01) | 6.3% (668) | 6.9% (718) | 7.3% (766) | 7.6% (791) | 8.4% (737) | 9.0% (756) |
| Saccharated iron oxide  (B03AC02) | 0.8% (82) | 0.8% (81) | 0.8% (81) | 0.7% (71) | 0.9% (78) | 0.9% (74) |
| Ferric oxide dextran complex (B03AC06) | 0.0%  (5) | 0.1%  (6) | 0.1%  (6) | 0.1% (12) | -%  (<5) | -%  (<5) |
| Ferric sodium gluconate complex (B03AC07) | 3.6% (379) | 3.4% (347) | 3.2% (336) | 2.8% (291) | 2.8% (243) | 3.0% (249) |
| Ferric derisomaltose  (B03AC08) | 0.3% (28) | 0.3% (32) | 0.2% (19) | 1.1% (113) | 1.4% (123) | 0.9% (72) |

ATC: Anatomical Therapeutic Chemical; ID/IDA: iron deficiency/iron deficiency anemia

##

## Supplementary Table 11

Healthcare resource utilization in subpopulations of patients with ID/IDA, 2021

|  | **Heart disease**  **(n = 44, 353)** | **Geriatric**  **(n = 40,662)** | **Menorr-hagia, other gynecol. Diseases**  **(n = 34,421)** | **Gastro-intestinal bleeding**  **(n = 20,417)** | **Inflamma-tory bowel disease**  **(n = 8,422)** |
| --- | --- | --- | --- | --- | --- |
| **Hospitalizations (all-cause)** |  |  |  |  |  |
| Patients ≥ 1 hospitalization, n (%) | 26,847 (60.3) | 24,329 (59.8) | 12,968 (37.7) | 12,494 (61.2) | 4,504 (53.5) |
| Admissions per patient, mean ± SD | 2.43 ± 1.88 | 2.30 ± 1.72 | 1.87 ± 1.50 | 2.59 ± 2.09 | 2.70 ± 2.24 |
| Length of stay (days), mean ± SD | 28.45 ± 38.99 | 27.00 ± 34.55 | 16.29 ± 36.48 | 28.81 ± 40.57 | 29.68 ± 44.80 |
| **Hospitalizations (due to ID/IDA)** |  |  |  |  |  |
| Patients ≥ 1 hospitalization, n (%) | 1,384 (3.1) | 1,401 (3.5) | 275 (0.8) | 778 (3.8) | 151 (1.8) |
| Admissions per patient, mean ± SD | 1.07 ± 0.46 | 1.05 ± 0.30 | 1.08 ± 0.28 | 1.07 ± 0.39 | 1.10 ± 0.47 |
| Length of stay (days), mean ± SD | 8.10 ± 9.32 | 7.87 ± 8.70 | 6.30 ± 8.17 | 8.19 ± 10.12 | 6.32 ± 6.18 |
| **Outpatient services (all-cause)** |  |  |  |  |  |
| Patients ≥ 1 utilization, n (%) | 44,483 (99.9) | 40,602 (99.9) | 34,421 (100) | 20,402 (99.9) | 8,420 (100) |
| Number per patient, mean ± SD | 40.60 ± 35.53 | 38.72 ± 32.47 | 30.05 ± 21.73 | 38.96 ± 32.29 | 37.69 ± 31.61 |
| **Outpatient services (due to ID/IDA)** |  |  |  |  |  |
| Patients ≥ 1 utilization, n (%) | 37,740 (84.7) | 34,159 (84.0) | 32,462 (94.3) | 17,463 (85.5) | 7,471 (88.7) |
| Number per patient, mean ± SD | 13.82 ± 22.68 | 13.73 ± 20.75 | 7.37 ± 9.07 | 11.78 ± 18.98 | 10.55 ± 17.56 |
| **Prescription drugs** |  |  |  |  |  |
| Patients ≥ 1 drug dispensation (any), n (%) | 43,811 (98.4) | 40,297 (99.1) | 31,862 (92.6) | 19,921 (97.6) | 8,119 (96.4) |
| Patients ≥ 1 drug dispensation (iron preparations), n, (%) | 16,420 (36.9) | 15,659 (38.5) | 10,735 (31.2) | 7,725 (37.8) | 2,977 (35.4) |
| **Sick leaves**, valid data sets, n | 44,458 | 40,562 | 34,394 | 20,396 | 8,414 |
| **Sick leave (all-cause)** |  |  |  |  |  |
| Patients ≥ 1 sick leave, n (%) | 3,921 (8.8) | <5 (-) | 10,594 (30.8) | 3,661 (18.0) | 2,445 (29.1) |
| Number per patient, mean ± SD | 2.54 ± 3.36 | - | 2.2 ± 1.75 | 2.74 ± 2.33 | 2.84 ± 2.27 |
| Length (days), mean ± SD | 66.22 ± 122.70 | - | 36.74 ± 80.13 | 54.44 ± 114.63 | 46.28 ± 96.02 |
| **Sick leave (due to ID/IDA)** |  |  |  |  |  |
| Patients ≥ 1 sick leave, n (%) | 59 (0.1) | 0 (0.0) | 191 (0.6) | 96 (0.5) | 48 (0.6) |
| Number per patient, mean ± SD | 1.59 ± 3.66 | - | 1.08 ± 0.34 | 1.43 ± 2.88 | 1.19 ± 0.53 |
| Length (days), mean ± SD | 36.85 ± 92.29 | - | 14.60 ± 29.45 | 20.92 ± 63.10 | 27.58 ± 83.30 |

ID/IDA: iron deficiency/iron deficiency anemia

## Supplementary Table 12

Expenditures associated with healthcare resource utilization in subpopulations of patients with ID/IDA, 2021

|  | Mean ± SD  cost per patient, in € | | | | |
| --- | --- | --- | --- | --- | --- |
|  | **Heart disease**  **(n = 44, 353)** | **Geriatric**  **(n = 40,662)** | **Menorrhagia, other gynecol. Diseases**  **(n = 34,421)** | **Gastrointestinal bleeding**  **(n = 20,417)** | **Inflammatory bowel disease**  **(n = 8,422)** |
| **Hospitalizations** |  |  |  |  |  |
| All-cause | 7,860 ± 15,695 | 7,326 ± 13,077 | 1,928 ± 5,934 | 7,820 ± 15,624 | 6,476 ± 15,630 |
| Due to ID/IDA | 114 ± 782 | 125 ± 802 | 24 ± 313 | 143 ± 912 | 57 ± 557 |
| **Outpatient services** |  |  |  |  |  |
| All-cause | 2,099 ± 4,625 | 1,910 ± 4,093 | 1,262 ± 1,821 | 1,841 ± 3,782 | 1,678 ± 3,457 |
| Due to ID/IDA | 704 ± 3,431 | 624 ± 2,968 | 248 ± 1,078 | 502 ± 2,609 | 416 ± 2,155 |
| **Prescription drug treatment** |  |  |  |  |  |
| Any drug treatment | 2,975 ± 10,482 | 2,755 ± 9,508 | 1,096 ± 6,709 | 3,572 ± 11,613 | 4,507 ± 11,473 |
| Iron preparations | 34 ± 120 | 34 ± 116 | 23 ± 90 | 44 ± 146 | 50 ± 157 |
| **Total cost** |  |  |  |  |  |
| All-cause^1^ | 12,934 ± 20,723 | 11,990 ± 17,783 | 4,286 ± 10,024 | 13,233 ± 21,138 | 12,661 ± 21,209 |
| ID/IDA-related^2^ | 853 ± 3,535 | 782 ± 3,087 | 294 ± 1,137 | 689 ± 2,781 | 523 ± 2,249 |
| **Sickness benefits** |  |  |  |  |  |
| All-cause | 156 ± 1,913 | - | 184 ± 1,861 | 228 ± 2,228 | 257 ± 2,215 |
| Due to ID/IDA | 1 ± 99 | - | 1 ± 79 | 1 ± 91 | - |

^1^sum of outpatient costs, costs for hospitalizations (all-cause), and costs for any prescription drug treatment

^2^sum of costs for hospitalizations due to ID/IDA, costs for outpatient services due to ID/IDA, and iron preparation

ID/IDA: iron deficiency/iron deficiency anemia; SD: standard deviation

## Supplementary Table 13

Healthcare resource utilization in the treatment cohort (subcohort: heart disease), 365 days before and after the index date

|  | Treatment subcohort: heart disease  (n= 231) | |
| --- | --- | --- |
|  | 365 days before the index date | 365 days after the index date |
| **Hospitalizations (all-cause)** |  |  |
| Patients ≥ 1 hospitalization, n (%) | 160 (69.3) | 153 (66.2) |
| Admissions per patient, mean ± SD | 2.99 ± 2.22 | 2.57 ± 1.93 |
| Length of stay (days), mean ± SD | 25.58 ± 30.60 | 21.30 ± 24.47 |
| **Hospitalization (due to ID/IDA)** |  |  |
| Patients ≥ 1 hospitalization, n (%) | 10 (4.3) | 7 (3.0) |
| Admissions per patient, mean ± SD | 1.00 ± 0.00 | 1.14 ± 0.38 |
| Length of stay (days), mean ± SD | 5.70 ± 3.65 | 6.14 ± 4.53 |
| **Outpatient services (all-cause)** |  |  |
| Patients ≥ 1 utilization, n (%) | 231 (100.0) | 230 (99.6) |
| Number per patient, mean ± SD | 50.56 ± 29.89 | 48.65 ± 36.38 |
| **Outpatient services (due to ID/IDA)** |  |  |
| Patients ≥ 1 utilization, n (%) | 215 (93.1) | 162 (70.1) |
| Number per patient, mean ± SD | 9.70 ± 8.97 | 14.18 ± 17.94 |
| **Prescription drugs** |  |  |
| Patients ≥ 1 drug dispensation (any), n (%) | 227 (98.3) | 231 (100.0) |
| Patients ≥ 1 drug dispensation (iron preparations), n (%) | 84 (36.4) | 231 (100.0) |
| **Sick leaves**, valid data sets, n | 231 | 231 |
| **Sick leave (all-cause)** |  |  |
| Patients ≥ 1 sick leave, n (%) | 37 (16.0) | 38 (16.5) |
| Number per patient, mean ± SD | 2.73 ± 1.88 | 2.24 ± 1.62 |
| Length (days), mean ± SD | 143.19 ± 198.17 | 130.08 ± 187.53 |
| **Sick leave (due to ID/IDA)** |  |  |
| Patients ≥ 1 sick leave, n (%) | <5 (-) | <5 (-) |
| Number per patient, mean ± SD | - | - |
| Length (days), mean ± SD | - | - |

ID/IDA: iron deficiency/iron deficiency anemia; SD: standard deviation

## Supplementary Table 14

Expenditures associated with healthcare resource utilization in the treatment cohort (subcohort: heart disease; n = 231), 365 days before and after the index date

|  | Mean cost ± SD  per patient, in 2021 Euro values | |
| --- | --- | --- |
|  | **365 days before the index date** | **365 days after the index date** |
| **Hospitalizations** |  |  |
| All-cause | 9,580 ± 16,233 € | 7,348 ± 14,461 € |
| Due to ID/IDA | 113 ± 582 € | 44 ± 366 € |
| **Outpatient services** |  |  |
| All-cause | 1,780 ± 2,289 € | 2,056 ± 3,566 € |
| Due to ID/IDA | 222 ± 246 € | 464 ± 2,445 € |
| **Prescription drug treatment** |  |  |
| Any drug treatment | 4,078 ± 7,609 € | 5,465 ± 8,977 € |
| Iron preparations | 53 ± 176 € | 303 ± 300 € |
| **Total cost** |  |  |
| All-cause^1^ | 15,439 ± 18,648 € | 14,870 ± 18,561 € |
| ID/IDA-related^2^ | 389 ± 663 € | 811 ± 2,518 € |
| **Sickness benefits** |  |  |
| All-cause | 395 ± 2,019 € | 432 ± 2,200 € |
| Due to ID/IDA | - | - |

^1^sum of outpatient costs, costs for hospitalizations (all-cause), and costs for any prescription drug treatment

^2^sum of costs for hospitalizations due to ID/IDA, costs for outpatient services due to ID/IDA, and iron preparations

ID/IDA: iron deficiency/iron deficiency anemia; SD: standard deviation

## Supplementary Table 15

Healthcare resource utilization in the treatment cohort (subcohort: geriatric), 365 days before and after the index date

|  | Treatment subcohort: geriatric  (n = 168) | |
| --- | --- | --- |
|  | 365 days before the index date | 365 days after the index date |
| **Hospitalizations (all-cause)** |  |  |
| Patients ≥ 1 hospitalization, n (%) | 115 (68.5) | 98 (58.3) |
| Admissions per patient, mean ± SD | 2.92 ± 2.14 | 2.92 ± 2.12 |
| Length of stay (days), mean ± SD | 26.80 ± 28.92 | 26.69 ± 25.58 |
| **Hospitalization (due to ID/IDA)** |  |  |
| Patients ≥ 1 hospitalization, n (%) | 10 (6.0) | 5 (3.0) |
| Admissions per patient, mean ± SD | 1.00 ± 0.00 | 1.20 ± 0.45 |
| Length of stay (days), mean ± SD | 5.10 ± 3.41 | 10.20 ± 6.72 |
| **Outpatient services (all-cause)** |  |  |
| Patients ≥ 1 utilization, n (%) | 168 (100.0) | 166 (98.8) |
| Number per patient, mean ± SD | 49.30 ± 26.31 | 45.86 ± 32.94 |
| **Outpatient services (due to ID/IDA)** |  |  |
| Patients ≥ 1 utilization, n (%) | 152 (90.5) | 114 (67.9) |
| Number per patient, mean ± SD | 11.10 ±9.96 | 14.83 ± 16.35 |
| **Prescription drugs** |  |  |
| Patients ≥ 1 drug dispensation (any), n (%) | 168 (100.0) | 168 (100.0) |
| Patients ≥ 1 drug dispensation (iron preparations), n (%) | 63 (37.5) | 168 (100.0) |
| **Sick leave,** valid data sets, n | 168 | 168 |
| **Sick leave (all-cause)** |  |  |
| Patients ≥ 1 sick leave, n (%) | 0 (0.0) | 0 (0.0) |
| Number per patient, mean ± SD | - | - |
| Length (days), mean ± SD | - | - |
| **Sick leave (due to ID/IDA)** |  |  |
| Patients ≥ 1 sick leave, n (%) | 0 (0.0) | 0 (0.0) |
| Number per patient, mean ± SD | - | - |
| Length (days), mean ± SD | - | - |

ID/IDA: iron deficiency/iron deficiency anemia

## Supplementary Table 16

Expenditures associated with healthcare resource utilization in the ferric maltol treatment cohort (subcohort: geriatric; n = 168), 365 days before and after the index date

|  | **Mean cost ± SD**  per patient, in 2021 Euro values | |
| --- | --- | --- |
|  | **365 days before the index date** | **365 days after the index date** |
| **Hospitalizations** |  |  |
| All-cause | 10,302 ± 17,264 € | 7,538 ± 13,225 € |
| Due to ID/IDA | 177 ± 743 € | 161 ±1,481 € |
| **Outpatient services** |  |  |
| All-cause | 1,789 ± 2,527 € | 1,969 ±3,427 € |
| Due to ID/IDA | 266 ± 334 € | 432 ±2,135 € |
| **Prescription drug treatment** |  |  |
| Any drug treatment | 3,541 ± 6,974 € | 4,492 ± 7,870 € |
| Iron preparations | 63 ± 201 € | 309 ± 315 € |
| **Total cost** |  |  |
| All-cause^1^ | 15,633 ± 19,354 € | 14,000 ± 17,058 € |
| ID/IDA-related^2^ | 506 ±855 € | 902 ±2,637 € |
| **Sickness benefits** |  |  |
| All-cause | - | - |
| Due to ID/IDA | - | - |

^1^sum of outpatient costs, costs for hospitalizations (all-cause), and costs for any prescription drug treatment

^2^sum of costs for hospitalizations due to ID/IDA, costs for outpatient services due to ID/IDA, and iron preparations

ID/IDA: iron deficiency/iron deficiency anemia; Q1, Q3: quartile 1 and 3

## Supplementary Table 17

Healthcare resource utilization in the ferric maltol treatment cohort (subcohort: menorrhagia and other gynecological diseases), 365 days before and after the index date

|  | Treatment subcohort: menorrhagia and other gynecological diseases (n= 215) | |
| --- | --- | --- |
|  | 365 days before the index date | 365 days after the index date |
| **Hospitalizations (all-cause)** |  |  |
| Patients ≥ 1 hospitalization, n (%) | 98 (45.6) | 107 (49.8) |
| Admissions per patient, mean ± SD | 2.46 ± 1.83 | 2.08 ± 1.54 |
| Length of stay (days), mean ± SD | 22.77 ± 41.04 | 23.41 ± 48.58 |
| **Hospitalization (due to ID/IDA)** |  |  |
| Patients ≥ 1 hospitalization, n (%) | 5 (2.3) | <5 (-) |
| Admissions per patient, mean ± SD | 1.40 ± 0.55 | - |
| Length of stay (days), mean ± SD | 5.00 ± 4.74 | - |
| **Outpatient services (all-cause)** |  |  |
| Patients ≥ 1 utilization, n (%) | 215 (100.0) | 215 (100.0) |
| Number per patient, mean ± SD | 40.52 ± 25.06 | 37.60 ± 27.12 |
| **Outpatient services (due to ID/IDA)** |  |  |
| Patients ≥ 1 utilization, n (%) | 210 (97.7) | 162 (75.4) |
| Number per patient, mean ± SD | 8.36 ± 7.80 | 9.42 ± 8.48 |
| **Prescription drugs** |  |  |
| Patients ≥ 1 drug dispensation (any), n (%) | 205 (95.4) | 215 (100.0) |
| Patients ≥ 1 drug dispensation (iron preparations), n (%) | 78 (36.3) | 215 (100.0) |
| **Sick leave,** valid data sets, n | 215 | 215 |
| **Sick leave (all-cause)** |  |  |
| Patients ≥ 1 sick leave, n (%) | 93 (43.3) | 91 (42.3) |
| Number per patient, mean ± SD | 2.72 ± 1.84 | 2.47 ± 1.76 |
| Length (days), mean ± SD | 75.39 ± 141.81 | 72.60 ± 132.41 |
| **Sick leave (due to ID/IDA)** |  |  |
| Patients ≥ 1 sick leave, n (%) | <5 (-) | <5 (-) |
| Number per patient, mean ± SD | - | - |
| Length (days), mean ± SD | - | - |

ID/IDA: iron deficiency/iron deficiency anemia

## Supplementary Table 18

Expenditures associated with healthcare resource utilization in the ferric maltol treatment cohort (subcohort: menorrhagia and other gynecological diseases; n = 215), 365 days before and after the index date

|  | Mean cost ± SD  per patient, in 2021 Euro values | |
| --- | --- | --- |
|  | **365 days before the index date** | **365 days after the index date** |
| **Hospitalizations** |  |  |
| All-cause | 3,129 ± 8,426 € | 2,307 ±4,954 € |
| Due to ID/IDA | 57 ± 428 € | 3 ± 41 € |
| **Outpatient services** |  |  |
| All-cause | 1,376 ± 1,278 € | 1,447 ± 1,469 € |
| Due to ID/IDA | 205 ± 199 € | 194 ± 234 € |
| **Prescription drug treatment** |  |  |
| Any drug treatment | 3,421 ±7,161 € | 5,017 ± 9,931 € |
| Iron preparations | 45 ±136 € | 243 ± 201 € |
| **Total cost** |  |  |
| All-cause^1^ | 7,926 ±12,157 € | 8,771 ± 11,917 € |
| ID/IDA-related^2^ | 307 ± 521 € | 439 ± 338 € |
| **Sickness benefits** |  |  |
| All-cause | 533 ± 2,339 € | 510 ± 2,229 € |
| Due to ID/IDA | - | - |

^1^sum of outpatient costs, costs for hospitalizations (all-cause), and costs for any prescription drug treatment

^2^sum of costs for hospitalizations due to ID/IDA, costs for outpatient services due to ID/IDA, and iron preparations

ID/IDA: iron deficiency/iron deficiency anemia

## Supplementary Table 19

Healthcare resource utilization in the ferric maltol treatment cohort (subcohort: gastrointestinal bleeding), 365 days before and after the index date

|  | Treatment subcohort: gastrointestinal bleeding  (n= 377) | |
| --- | --- | --- |
|  | 365 days before the index date | 365 days after the index date |
| **Hospitalizations (all-cause)** |  |  |
| Patients ≥ 1 hospitalization, n (%) | 219 (58.1) | 188 (49.9) |
| Admissions per patient, mean ± SD | 2.74 ± 2.30 | 2.68 ± 2.04 |
| Length of stay (days), mean ± SD | 23.63 ± 37.78 | 26.45 ±52.61 |
| **Hospitalization (due to ID/IDA)** |  |  |
| Patients ≥ 1 hospitalization, n (%) | 11 (2.9) | 5 (1.3) |
| Admissions per patient, mean ± SD | 1.27 ± 0.47 | 1.20 ± 0.45 |
| Length of stay (days), mean ± SD | 5.18 ± 4.33 | 8.60 ± 8.08 |
| **Outpatient services (all-cause)** |  |  |
| Patients ≥ 1 utilization, n (%) | 377 (100.0) | 375 (99.5) |
| Number per patient, mean ± SD | 44.23 ± 24.45 | 39.97 ± 28.03 |
| **Outpatient services (due to ID/IDA)** |  |  |
| Patients ≥ 1 utilization, n (%) | 366 (97.1) | 253 (67.1) |
| Number per patient, mean ± SD | 8.69 ± 8.50 | 10.42 ± 8.83 |
| **Prescription drugs** |  |  |
| Patients ≥ 1 drug dispensation (any), n (%) | 374 (99.2) | 377 (100.0) |
| Patients ≥ 1 drug dispensation (iron preparations), n (%) | 137 (36.2) | 377 (100.0) |
| **Sick leave,** valid data sets, n | 377 | 377 |
| **Sick leave (all-cause)** |  |  |
| Patients ≥ 1 sick leave, n (%) | 154 (40.9) | 141 (37.4) |
| Number per patient, mean ± SD | 3.02 ± 2.10 | 2.70 ± 2.08 |
| Length (days), mean ± SD | 84.10 ± 140.22 | 82.13 ± 136.52 |
| **Sick leave (due to ID/IDA)** |  |  |
| Patients ≥ 1 sick leave, n (%) | 8 (2.1) | <5 (-) |
| Number per patient, mean ± SD | 1.25 ± 0.46 | - |
| Length (days), mean ± SD | 47.88 ± 88.29 | - |

ID/IDA: iron deficiency/iron deficiency anemia

## Supplementary Table 20

Expenditures associated with healthcare resource utilization in the ferric maltol treatment cohort (subcohort: gastrointestinal bleeding; n = 377), 365 days before and after the index date

|  | Mean cost ± SD  per patient, in 2021 Euro values | |
| --- | --- | --- |
|  | **365 days before the index date** | **365 days after the index date** |
| **Hospitalizations** |  |  |
| All-cause | 5,373 ± 11,808 € | 4,134 ± 10,920 € |
| Due to ID/IDA | 74 ± 493 € | 65 ± 975 € |
| **Outpatient services** |  |  |
| All-cause | 1,509 ± 1,805 € | 1,493 ± 2,017 € |
| Due to ID/IDA | 237 ± 276 € | 201 ± 280 € |
| **Prescription drug treatment** |  |  |
| Any drug treatment | 7,888 ± 12,362 € | 9,931 ± 12,881 € |
| Iron preparations | 67 ± 181 € | 306 ± 321 € |
| **Total cost** |  |  |
| All-cause^1^ | 14,768 ± 16,768 € | 15,558 ± 17,478 € |
| ID/IDA-related^2^ | 379 ± 3,178 € | 571 ± 1,094 € |
| **Sickness benefits** |  |  |
| All-cause | 739 ± 3,178 € | 472 ± 1,990 € |
| Due to ID/IDA | - | - |

^1^sum of outpatient costs, costs for hospitalizations (all-cause), and costs for any prescription drug treatment

^2^sum of costs for hospitalizations due to ID/IDA, costs for outpatient services due to ID/IDA, and iron preparations

ID/IDA: iron deficiency/iron deficiency anemia; SD: standard deviation

## Supplementary Table 21

Healthcare resource utilization in the treatment cohort (subcohort: inflammatory bowel disease), 365 days before and after the index date

|  | Treatment subcohort: inflammatory bowel disease (n= 304) | |
| --- | --- | --- |
|  | 365 days before the index date | 365 days after the index date |
| **Hospitalizations (all-cause)** |  |  |
| Patients ≥ 1 hospitalization, n (%) | 168 (55.3) | 145 (47.7) |
| Admissions per patient, mean ± SD | 2.66 ± 2.20 | 2.61 ± 1.76 |
| Length of stay (days), mean ± SD | 23.02 ± 40.03 | 28.25 ± 58.45 |
| **Hospitalization (due to ID/IDA)** |  |  |
| Patients ≥ 1 hospitalization, n (%) | 7 (2.3) | <5 (-) |
| Admissions per patient, mean ± SD | 1.43 ± 0.53 | - (-) |
| Length of stay (days), mean ± SD | 4.14 ± 4.02 | - (-) |
| **Outpatient services (all-cause)** |  |  |
| Patients ≥ 1 utilization, n (%) | 304 (100.0) | 303 (99.7) |
| Number per patient, mean ± SD | 44.06 ± 23.53 | 39.97 ± 26.73 |
| **Outpatient services (due to ID/IDA)** |  |  |
| Patients ≥ 1 utilization, n (%) | 296 (97.4) | 205 (67.4) |
| Number per patient, mean ± SD | 8.19 ± 7.94 | 9.75 ± 8.43 |
| **Prescription drugs** |  |  |
| Patients ≥ 1 drug dispensation (any), n (%) | 303 (99.7) | 304 (100.0) |
| Patients ≥ 1 drug dispensation (iron preparations), n (%) | 106 (34.9) | 304 (100.0) |
| **Sick leave,** valid data sets, n | 304 | 304 |
| **Sick leave (all-cause)** |  |  |
| Patients ≥ 1 sick leave, n (%) | 137 (45.1) | 126 (41.5) |
| Number per patient, mean ± SD | 3.04 ± 2.13 | 2.80 ± 2.15 |
| Length (days), mean ± SD | 80.82 ± 135.94 | 76.79 ± 130.39 |
| **Sick leave (due to ID/IDA)** |  |  |
| Patients ≥ 1 sick leave, n (%) | 8 (2.6) | <5 (-) |
| Number per patient, mean ± SD | 1.25 ± 0.46 | - (-) |
| Length (days), mean ± SD | 47.88 ± 88.29 | - (-) |

ID/IDA: iron deficiency/iron deficiency anemia; SD: standard deviation

## Supplementary Table 22

Expenditures associated with healthcare resource utilization in the ferric maltol treatment cohort (subcohort: inflammatory bowel disease; n = 304), 365 days before and after the index date

|  | **Mean cost ± SD**  per patient, in 2021 Euro values | |
| --- | --- | --- |
|  | **365 days before the index date** | **365 days after the index date** |
| **Hospitalizations** |  |  |
| All-cause | 3,890 ± 8,367 € | 3,684 ± 10,875 € |
| Due to ID/IDA | 45 ± 356 € | 66 ± 1,061 € |
| **Outpatient services** |  |  |
| All-cause | 1,420 ± 1,017 € | 1,358 ± 1,150 € |
| Due to ID/IDA | 222 ± 229 € | 187 ± 247 € |
| **Prescription drug treatment** |  |  |
| Any drug treatment | 9,177 ± 13,134 € | 11,752 ± 13,622 € |
| Iron preparations | 66 ± 185 € | 316 ± 338 € |
| **Total cost** |  |  |
| All-cause^1^ | 14,487 ± 15,348 € | 16,794 ± 18,005€ |
| ID/IDA-related^2^ | 334 ± 489 € | 569 ± 1,170 € |
| **Sickness benefits** |  |  |
| All-cause | 806 ± 3,296 € | 493 ± 2,093 € |
| Due to ID/IDA | - | - |

^1^sum of outpatient costs, costs for hospitalizations (all-cause), and costs for any prescription drug treatment

^2^sum of costs for hospitalizations due to ID/IDA, costs for outpatient services due to ID/IDA, and iron preparations

ID/IDA: iron deficiency/iron deficiency anemia; SD: standard deviation

## Supplementary Table 23

Treatments of patients with ID/IDA of the ferric maltol treatment cohort (subcohort: heart disease; n = 231) during the year before and after treatment initiation at index date

| Treatment option | Treatment subcohort: Heart disease (n = 231) | |
| --- | --- | --- |
|  | 1 year before index  n (%) | 1 year after index  n (%) |
| No treatment with iron preparations | 147 (63.6%) | 70 (30.3%) |
| Any treatment with iron preparations | 84 (36.4%) | 161 (69.7%) |
| thereof, treatment with bivalent oral iron preparations | 59 (70.2%) | 34 (21.1%) |
| thereof, treatment with ferric maltol | 0 (0.0%) | 111 (68.9%) |
| thereof, treatment with parenteral iron preparations | 34 (40.5%) | 45 (28.0%) |

ID/IDA: iron deficiency/iron deficiency anemia; no patients received trivalent ferric oxide polymaltose in the year before and after the index date

## Supplementary Table 24

Treatments of patients with ID/IDA of the ferric maltol treatment cohort (subcohort: geriatric; n = 168) during the year before and after treatment initiation at index date

| Treatment option | Treatment subcohort: Geriatric (n = 168) | |
| --- | --- | --- |
|  | 1 year before index  n (%) | 1 year after index  n (%) |
| No treatment with iron preparations | 105 (62.5%) | 52 (31.0%) |
| Any treatment with iron preparations | 63 (37.5%) | 116 (69.1%) |
| thereof, treatment with bivalent oral iron preparations | 41 (65.1%) | 24 (20.7%) |
| thereof, treatment with ferric maltol | 0 (0.0%) | 88 (75.9%) |
| thereof, treatment with parenteral iron preparations | 27 (42.9%) | 25 (21.6%) |

ID/IDA: iron deficiency/iron deficiency anemia; no patients received trivalent ferric oxide polymaltose in the year before and after the index date

## Supplementary Table 25

Treatments of patients with ID/IDA of the ferric maltol treatment cohort (subcohort: Menorrhagia and other gynecological diseases; n = 215) during the year before and after treatment initiation at index date

| Treatment option | Treatment subcohort: Menorrhagia and other gynecological diseases (n = 215) | |
| --- | --- | --- |
|  | 1 year before index  n (%) | 1 year after index  n (%) |
| No treatment with iron preparations | 137 (63.7%) | 80 (37.2%) |
| Any treatment with iron preparations | 78 (36.3%) | 135 (62.8%) |
| thereof, treatment with bivalent oral iron preparations | 45 (57.7%) | 18 (13.3%) |
| thereof, treatment with ferric maltol | 0 (0.0%) | 91 (67.4%) |
| thereof, treatment with parenteral iron preparations | 41 (52.6%) | 52 (38.5%) |

ID/IDA: iron deficiency/iron deficiency anemia; no patients received trivalent ferric oxide polymaltose in the year before and after the index date

## Supplementary Table 26

Treatments of patients with ID/IDA of the ferric maltol treatment cohort (subcohort: Gastrointestinal bleeding; n = 377) during the year before and after treatment initiation at index date

| Treatment option | Treatment subcohort: Gastrointestinal bleeding (n = 377) | |
| --- | --- | --- |
|  | 1 year before index  n (%) | 1 year after index  n (%) |
| No treatment with iron preparations | 240 (63.7%) | 128 (34.0%) |
| Any treatment with iron preparations | 137 (36.3%) | 249 (66.1%) |
| thereof, treatment with bivalent oral iron preparations | 75 (54.7%) | 30 (12.0%) |
| thereof, treatment with ferric maltol | 0 (0.0%) | 167 (67.1%) |
| thereof, treatment with parenteral iron preparations | 75 (54.7%) | 102 (41.0%) |

ID/IDA: iron deficiency/iron deficiency anemia; no patients received trivalent ferric oxide polymaltose in the year before and after the index date

## Supplementary Table 27

Treatments of patients with ID/IDA of the ferric maltol treatment cohort (subcohort: Inflammatory bowel disease; n = 304) during the year before and after treatment initiation at index date

| Treatment option | Treatment subcohort: Inflammatory bowel disease (n = 304) | |
| --- | --- | --- |
|  | 1 year before index  n (%) | 1 year after index  n (%) |
| No treatment with iron preparations | 198 (65.1%) | 108 (35.5%) |
| Any treatment with iron preparations | 106 (34.9%) | 196 (64.5%) |
| thereof, treatment with bivalent oral iron preparations | 53 (50.0%) | 14 (7.1%) |
| thereof, treatment with ferric maltol | 0 (0.0%) | 131 (66.8%) |
| thereof, treatment with parenteral iron preparations | 62 (58.5%) | 92 (46.9%) |

ID/IDA: iron deficiency/iron deficiency anemia; no patients received trivalent ferric oxide polymaltose in the year before and after the index date
